# Characterizing Social Media Metrics of Scholarly Papers: The Effect of Document Properties and Collaboration Patterns

**DOI:** 10.1371/journal.pone.0120495

**Published:** 2015-03-17

**Authors:** Stefanie Haustein, Rodrigo Costas, Vincent Larivière

**Affiliations:** 1 École de bibliothéconomie et des sciences de l’information, Université de Montréal, C.P. 6128, Succ. Centre-Ville, Montréal, Canada; 2 Center for Science and Technology Studies, Leiden University, Wassenaarseweg, Leiden, The Netherlands; 3 Observatoire des Sciences et des Technologies (OST), Centre Interuniversitaire de Recherche sur la Science et la Technologie (CIRST), Université du Québec à Montréal, CP 8888, Succ. Centre-Ville, Montréal, Canada; Katholieke Universiteit Leuven, BELGIUM

## Abstract

A number of new metrics based on social media platforms—grouped under the term “altmetrics”—have recently been introduced as potential indicators of research impact. Despite their current popularity, there is a lack of information regarding the determinants of these metrics. Using publication and citation data from 1.3 million papers published in 2012 and covered in Thomson Reuters’ Web of Science as well as social media counts from Altmetric.com, this paper analyses the main patterns of five social media metrics as a function of document characteristics (i.e., discipline, document type, title length, number of pages and references) and collaborative practices and compares them to patterns known for citations. Results show that the presence of papers on social media is low, with 21.5% of papers receiving at least one tweet, 4.7% being shared on Facebook, 1.9% mentioned on blogs, 0.8% found on Google+ and 0.7% discussed in mainstream media. By contrast, 66.8% of papers have received at least one citation. Our findings show that both citations and social media metrics increase with the extent of collaboration and the length of the references list. On the other hand, while editorials and news items are seldom cited, it is these types of document that are the most popular on Twitter. Similarly, while longer papers typically attract more citations, an opposite trend is seen on social media platforms. Finally, contrary to what is observed for citations, it is papers in the Social Sciences and humanities that are the most often found on social media platforms. On the whole, these findings suggest that factors driving social media and citations are different. Therefore, social media metrics cannot actually be seen as alternatives to citations; at most, they may function as complements to other type of indicators.

## Introduction

The measurement of scientific publications and citations has a long tradition. As far back as the early 20th century, scholars studied the characteristics of scientific papers and their bibliographies, mainly in a library management context [1,2], measuring the research productivity of researchers [3], the frequency of words in texts [4] and the scattering of scientific literature [5]; these landmark papers formed the basic laws of bibliometrics. The creation of the Science Citation Index in the 1960s [6] stimulated research on the relationship between citations and papers’ characteristics, such as their collaboration [7], length [8], or number of references [9]. Theories have also been suggested to explain the citation process, which provided a framework for the use of citations in research evaluation and information retrieval [10–15]. Subsequently, in the natural and medical sciences, bibliometric indicators are generally recognized as indicators of scientific activity and impact, despite continuous debates on their validity and usefulness in a research evaluation context [16–19]. The knowledge drawn from these studies has helped to understand publication and citation behavior and has informed the construction of appropriate bibliometric indicators.

More recently, with the advent of social media in scholarly communication, a spectrum of social media-based metrics—generally regrouped under the umbrella term “altmetrics”—have been proposed as alternatives to citation-based indicators [20–22]. Embedded in the social web, this new family of indicators has the ambition, in the eyes of many, to estimate scientific impact much faster than citations, and to measure the impact of scientific discoveries outside the scientific community. There is an important ongoing discussion around them, ranging from conceptual issues and terminology [23] to their actual signification and usefulness [24–27]. So far, most of the studies have focused on determining the amount of papers that receive attention (i.e., have been covered) on the various platforms [27–29] as well as the correlation between citations and various social media event counts, with the aim of determining whether both types of indicators measure similar concepts [28,30,31]. Most studies show that, although citations and the new metrics are to some extent positively correlated, these correlations are very weak—except for Mendeley, which shows a moderate positive correlation with citations [31–33]. Despite this correlation, it has become clear that social medial metrics and citations indicate different aspects of the diffusion and use of scientific documents, and that the former cannot be considered a replacement of the latter [28,30,34]. For example, papers with funny titles, curious topics and “the usual trilogy of sex, drugs, and rock and roll” [35] are among the most popular on Twitter. Whether various social media platforms can serve as sources of valid indicators of the broader impact of scientific papers remains to be proven.

Since previous research has mostly focused on the relationship between social media mentions and citations, little is known about the factors influencing social media counts. The goal of this paper is to contribute to this understanding by exploring, by scientific domain, the relationship between scientific papers’ characteristics—such as number of pages and references, title length and collaboration patterns—and the different social media metrics. More specifically, the paper aims at answering the following research questions:

- What is the prevalence—as measured through coverage, density and intensity—of social and mainstream media mentions of papers across scientific fields and how does it vary by document type?
- How are various document characteristics—such as number of pages and references, title length and number of authors, institutions and countries—influencing social media mentions and citations?
- Which type of documents—articles, reviews, news items, editorials, etc.—receive the most attention on Twitter, Facebook, Google+, blogs and in mainstream media?

Social and mainstream media metrics analyzed in this paper include scientific blogs, Twitter, Facebook, Google+ and mainstream media and newspaper mentions, as covered by Altmetric.com. By combining these various social media sources with traditional bibliometric indicators, this paper aims to perform the first large-scale characterization of the drivers of social media metrics and to contrast them with the patterns observed for citations—the latter being much better known. Comparing these patterns to what is already known about citations will aid in the understanding of these new metrics and, it is hoped, to guide much needed qualitative studies on user motivations and the various processes behind these counts.

## Materials and Methods

### Databases

Publication and citation data was drawn from Thomson Reuters’ Web of Science (WoS), which includes the *Science Citation Index Expanded*, the *Social Science Citation Index* and the *Arts and Humanities Citation Index*. These three databases annually index documents published in about 12,000 journals, covering all areas of research. The disciplinary classifications used in this paper are those of the *Leiden Ranking (LR) 2013*, which places journal-based WoS subject categories into five main scientific disciplines. An important characteristic of this classification is that publications in general multidisciplinary journals such as Nature, PNAS, and Science are assigned to a specific field based on their referencing behavior, avoiding a problematic heterogeneous multidisciplinary category. In order to study the differences across document types, we have used the WoS document type classification. Although imperfect—particularly when it comes to distinguishing standard articles from reviewarticles [36]—this classification has the advantage of being homogeneous across all disciplines and being easily applicable to our dataset. We also identified retracted papers and retraction notices by retrieving papers with titles that respectively begin with or included the strings “retracted” or “retraction”, as this is how these documents are labeled in WoS.

Only papers published in 2012 were considered (N = 1,339,279), as this year provides the best compromise between the length of the citation window—citations to papers take time to accumulate—and the recent uptake of social media activity [25]. Citations to 2012 papers were counted until the end of 2013, which allows for a citation window of at least one complete year. Selecting 2012 publications also has the advantage of guaranteeing complete coverage of social media data for the whole year, as Altmetric.com started data collection mid-2011 [30].

Altmetric.com was chosen as the data source for social media and mainstream media counts, as it is the most comprehensive source of social media data associated with scientific papers [29]. The exception to this is Mendeley, for which Altmetric.com covers readership counts only for publications that are mentioned in at least one of the other covered social media platforms, thus underestimating Mendeley coverage. Because of this limitation, Mendeley data has been excluded from this analysis. Altmetric.com data includes counts collected up to 18/10/2013. Given the quick uptake of social media-based indicators (excluding Mendeley) reported by Thelwall et al. [25], we consider that the social media activity window of nearly a full year considered in this study is long enough to cover the vast majority of social media activity around papers published in 2012.

As the Digital Object Identifier (DOI) was used as the linkage between WoS and Altmetric.com data, publications are limited to those with a DOI as recorded in WoS. As one might expect, the DOIs are not available evenly across scientific disciplines. While, for most fields, the proportion of journals with publications with a DOI is very high (e.g. higher than 70% of all journals in most fields, see Supporting Information, S1 Fig), a substantial share of journals (30%), particularly in the Social Sciences and Humanities, do not use DOIs as a standard document id. Hence, for papers published in the latter group of journals, results from Altmetric.com are more likely to underestimate their actual online visibility, which represents a limitation of this study (as well as the great majority of social media metrics analyses).

### Indicators and variables

Several indicators are used in this paper comparing traditional publication and citations indicators with social media counts. While document characteristics and collaboration indicators are considered as independent variables, citations, blog posts, Twitter users, Facebook, Google+ shares and mainstream media mentions serve as dependent variables. All of these metrics have been compiled at the paper-level and are presented below with abbreviations provided in square brackets.

### Dependent variables

- Citations
  - Number of citations [C]: absolute number of citations received by publications until the end of 2013, including self-citations. We considered absolute instead of normalized citations for better comparison with social media counts.
  - Number of self-citations [SC]: number of self-citations received by publications until the end of 2013. A self-citation is counted when the cited and the citing paper have at least one author in common.
- Social and mainstream media counts (more information on the data extraction by Altmetric.com can be found in http://www.altmetric.com/sources.php)
  - Scholarly blog posts monitored by Altmetric.com [B];
  - Number of unique Twitter users [T];
  - Public Facebook shares [F]: number of public mentions by Facebook users;
  - Google+ mentions [G];
  - News and mainstream media outlets [M].

### Independent variables

- Document characteristics
  - Scientific discipline according to the Leiden Ranking classification [LR];
  - Document type [DT];
  - Number of pages [PG]: absolute number of pages of a publication derived from the beginning and end page of a document. Absolute numbers of pages were used instead of normalized value in order to present easily interpretable values. In any case, in future research normalized approaches should be included in order to explore this factor more thoroughly;
  - Number of references [NR]: number of cited sources in the reference list, this includes all cited documents including non-source items;
  - Number of characters in title [TI]: length of a document’s title in the number of characters.
- Collaboration
  - Number of authors [AU]: number of authors that signed a document;
  - Number of institutions [IN]: number of distinct institutions to which authors of the document were affiliated;
  - Number of countries [CU]: number of distinct countries found in the authors’ addresses.

The prevalence of social media metrics is measured through coverage, density and intensity of the respective metrics. Coverage is defined as the percentage of papers with at least one social media event or citation. Density is the average number of social media counts or citations per paper (i.e. considering all publications included in the study), while intensity indicates the average number of social media or citation counts for all documents with at least one event (non-zero counts). While coverage reflects the probability of a document to be cited or mentioned on the particular platform, the intensity rate aims to measure the frequency or popularity with which documents are (re-)used once they are on the platform. Density indicates the average popularity across all documents and is heavily influenced by the uptake of the particular social media platform as well as the ability of Altmetric.com to capture counts, whereas the intensity indicator remains independent of the coverage and zero values. All three indicators should be considered in order to explain the differences between citations and various social media indicators. In addition to these three indicators, we calculated Spearman correlations between all metrics based on all documents as well as correlations between citations and blog, Twitter, Facebook, Google+ and mainstream media counts, excluding documents with respective zero counts for each indicator. Simple linear regression analyses were performed for citations and social and mainstream media counts as a function of the number of pages, references, number of characters in the title, authors, institutions and countries.

## Results and Discussion

### Scientific Disciplines

Table 1 presents density (i.e. mean event per paper) and coverage (i.e. percentage of papers with at least one event) for social and mainstream media counts and citations as well as documents characteristics and collaboration indicators for all 1,399,279 documents in WoS and per LR field. It shows that the social media platform with the greatest prevalence is Twitter, with 21.5% of all publications tweeted at least once and 0.78 times on average. The values for the other social media counts are extremely small with coverage values below 5% and density below 0.1 in all cases. Twitter is followed by Facebook, blogs, Google+ and mainstream media in terms of density and coverage. Due to the low uptake of social media activity associated with these documents, the citation density (i.e., the average citation rate, 3.17) is much higher than any of the social media metrics. In fact, two-thirds of all 2012 papers had received at least one citation by the end of 2013, this being a sign of the stronger prevalence of citations over social media metrics across scientific publications, quite contrary to the belief that social media metrics can overcome citation delay.

**Table 1:** Density and coverage per LR field.

Leiden Ranking field		Document characteristics			Collaboration			Citations		Social and mainstream media counts				
		PG	NR	TI	AU	IN	CU	C	SC	B	T	F	G	M
Total, N = 1,339,279	Density	9.26	36.06	96.45	2.13	5.15	1.32	3.17	0.81	0.03	0.78	0.08	0.01	0.01
	*Std*. *dev*.	*7*.*60*	*31*.*47*	*36*.*39*	*3*.*58*	*35*.*81*	*0*.*95*	*8*.*02*	*1*.*70*	*0*.*29*	*7*.*03*	*1*.*16*	*0*.*30*	*0*.*17*
	Coverage	*n/c*						66.8	*n/c*	1.9	21.5	4.7	0.8	0.7
Biomedical & health sciences, N = 595,254	Density	7.73	35.96	100.91	2.32	5.55	1.31	3.57	0.75	0.03	1.28	0.14	0.02	0.01
	*Std*. *dev*.	*6*.*73*	*33*.*08*	*38*.*37*	*2*.*38*	*4*.*96*	*0*.*84*	*8*.*98*	*1*.*63*	*0*.*32*	*8*.*71*	*1*.*57*	*0*.*33*	*0*.*19*
	Coverage	*n/c*						70.1	*n/c*	2.1	31.7	7.5	1.0	0.7
Life & earth sciences, N = 254,817	Density	9.88	43.70	105.13	2.25	4.85	1.40	3.44	0.96	0.05	1.03	0.11	0.02	0.02
	*Std*. *dev*.	*6*.*67*	*31*.*65*	*35*.*12*	*1*.*78*	*4*.*24*	*0*.*83*	*7*.*34*	*1*.*73*	*0*.*44*	*9*.*53*	*1*.*15*	*0*.*33*	*0*.*29*
	Coverage	*n/c*						74.3	*n/c*	2.9	21.6	5.7	1.0	1.3
Mathematics & computer science, N = 135,445	Density	12.74	27.39	81.37	1.81	3.18	1.31	1.70	0.56	0.01	0.36	0.03	0.01	0.01
	*Std*. *dev*.	*8*.*48*	*19*.*58*	*28*.*20*	*1*.*16*	*2*.*79*	*0*.*61*	*4*.*77*	*1*.*29*	*0*.*24*	*5*.*68*	*0*.*58*	*0*.*39*	*0*.*15*
	Coverage	*n/c*						54.6	*n/c*	0.8	7.5	1.5	0.4	0.3
Natural sciences & engineering, N = 413,862	Density	8.78	35.39	97.22	2.11	6.33	1.35	3.83	1.13	0.03	0.34	0.03	0.01	0.01
	*Std*. *dev*.	*6*.*61*	*29*.*22*	*33*.*57*	*5*.*65*	*64*.*07*	*1*.*24*	*9*.*07*	*2*.*09*	*0*.*27*	*5*.*16*	*0*.*50*	*0*.*32*	*0*.*19*
	Coverage	*n/c*						73.7	*n/c*	1.8	12.9	2.3	0.6	0.8
Social sciences & humanities, N = 159,389	Density	12.62	37.56	82.18	1.69	2.47	1.21	1.43	0.35	0.05	1.33	0.10	0.02	0.01
	*Std*. *dev*.	*8*.*79*	*32*.*16*	*33*.*24*	*1*.*29*	*2*.*31*	*0*.*59*	*3*.*33*	*0*.*92*	*0*.*44*	*13*.*86*	*1*.*55*	*0*.*39*	*0*.*22*
	Coverage	*n/c*						45.8	*n/c*	2.7	26.0	4.8	1.1	0.9

Density (mean and standard deviation) and coverage (%) for document characteristics, collaboration, citations and social and mainstream media counts by LR fields.

Twitter density is higher in Social Sciences (1.33), Biomedical and Health Sciences (1.28), as well as Life and Earth Sciences (1.03), but very low in Mathematics and Computer Science (0.36) and Natural Sciences and Engineering (0.34). Similar differences across fields were also observed for 2011 publications [30]. These findings suggest a certain tendency towards some topics and fields of research to enjoy greater popularity among social media users as compared to others. It could be argued that disciplines that have stronger ties to society (e.g. Social Sciences and Humanities) or deal with specific concerns of people’s everyday lives (health or environmental problems; i.e., Biomedical and Health Sciences and Life and Earth Sciences) have a higher probability of appearing on social media platforms than publications from more technical and applied disciplines (e.g. Natural Sciences and Engineering) or with a higher technical and complexity component (e.g. Mathematics and Computer Science). A simple explanation might be that the general public is more able to understand and relate to “softer” science topics than complex technical issues from Physics, as the latter generally makes use of a very formal language. Another explanation might be that researchers from Natural Sciences, Engineering, Computer Science and Mathematics are less active on Twitter.

Similar patterns can be observed for public Facebook posts which are most frequent in Biomedical and Health Sciences (density = 0.14, coverage 7.5%) and blogs, which appear more frequently in Life and Earth Sciences (2.9% coverage) and Social Sciences and Humanities (2.7%), and are hardly visible for papers in Mathematics and Computer Sciences (0.8%). Google+ posts occur sparsely, with slightly higher prevalence in Social Sciences and Humanities (1.1% coverage). Mainstream media and news seem to show the least pronounced differences between disciplines, although Mathematics and Computer Science remains the LR discipline with the lowest media coverage (0.3%). Finally, results for document types and collaboration indicators show known patterns; for example, reference lists are significantly longer in Life and Earth Sciences (43.7) than in Mathematics and Computer Science (27.4), and the number of authors is the smallest in Social Sciences and Humanities (1.7). Relationships between these characteristics and social media metrics will be analyzed below.

### Document types

Table 2 provides an overview of the coverage (C), density (D) and intensity (I) of different metrics across document types to explore whether certain types of publications types attract greater audiences or are more popular on the various platforms. Less important document types (i.e., occurring less than 2,300 times, 0.03% of 1,339,279) are not shown. While coverage and density reflect the percentage of papers with at least one count and the average number of counts per paper as shown in Table 1 above, intensity measures the average number of counts for documents excluding zero values and thus reflects the rate with which documents are (re-)used once they are on the platform independently of the coverage.

**Table 2:** Prevalance of citations and social media metrics per document type.

		all	Article	Biographical Item	Book Review	Correction	Editorial Material	Letter	Meeting Abstract	News Item	Review
*N*		*1*,*338*,*885*	*1*,*132*,*428*	*2*,*302*	*21*,*710*	*9*,*817*	*60*,*533*	*29*,*410*	*13*,*071*	*4*,*880*	*64*,*734*
*%*		*99*.*97*	*84*.*56*	*0*.*17*	*1*.*62*	*0*.*73*	*4*.*52*	*2*.*20*	*0*.*98*	*0*.*36*	*4*.*83*
Citations	C	66.86	71.06	6.52	1.03	11.94	36.28	34.19	3.08	28.11	85.08
	D	3.17	3.24	0.10	0.01	0.22	1.05	0.80	0.04	0.72	7.48
	I	4.74	4.55	1.48	1.10	1.83	2.90	2.33	1.17	2.55	8.80
Blogs	C	1.86	1.88	0.70	0.14	2.19	2.34	0.82	0.03	2.23	2.51
	D	0.03	0.03	0.01	0.00	0.02	0.03	0.01	0.00	0.03	0.03
	I	1.51	1.53	1.00	1.00	1.09	1.48	1.49	1.00	1.19	1.31
Twitter	C	21.50	20.99	13.03	5.16	10.33	27.34	18.16	2.09	42.48	36.17
	D	0.78	0.74	0.34	0.09	0.18	1.59	0.45	0.04	3.02	1.38
	I	3.65	3.50	2.62	1.83	1.76	5.81	2.46	1.93	7.10	3.81
Facebook	C	4.70	4.49	2.74	1.24	1.29	7.41	3.36	0.22	7.36	8.80
	D	0.08	0.08	0.03	0.01	0.01	0.15	0.05	0.00	0.12	0.16
	I	1.78	1.77	1.25	1.07	1.03	1.98	1.35	1.03	1.57	1.84
Google+	C	0.75	0.72	0.26	0.04	0.19	1.25	0.44	0.02	3.85	1.34
	D	0.01	0.01	0.00	0.00	0.00	0.02	0.01	0.00	0.04	0.02
	I	1.66	1.70	1.17	1.00	1.16	1.64	1.49	1.00	1.11	1.39
Mainstream media	C	0.69	0.72	0.00	0.00	0.07	0.82	0.19	0.01	0.37	0.61
	D	0.01	0.01	0.00	0.00	0.00	0.01	0.00	0.00	0.01	0.01
	I	1.54	1.57	n/a	n/a	1.14	1.32	2.05	1.00	1.44	1.25

Coverage (C): percentage of document types with at least one citation, tweet, blog, mainstream media, Facebook or Google+ mention. Density (D): average number of events per paper for all papers and Intensity (I): average number of events per paper for those with at least one event. Document types which occurred less than 2,300 times in 2012 were excluded from the analysis. (Excluded document types include Poetry (165 papers), Reprint (66), Art Exhibit Review (35), Software Review (31), Bibliography (29), Theater Review (21), Record Review (14), Fiction, Creative Prose (10), Film Review (8), Music Score Review (6), TV Review, Radio Review (2), Database Review (1), Abstract of Published Item (1), Excerpt (1), Music Performance Review (1) and Music Score (1). For an explanation of WoS document types see http://images.webofknowledge.com/WOKRS59B4/help/WOS/hs_document_type.html.)

As to be expected, Table 2 shows that research articles make up the vast majority of document types (84.6%), followed by reviews (4.8%), editorials (4.5%), letters (2.2%) and book reviews (1.6%). As already known from bibliometric studies [37], reviews are on average cited much more than any other document type (7.48), and low citedness rates (i.e. the percentage of documents with citations) can be observed for book reviews (1.03%), meeting abstracts (3.08%), biographical items (6.52%), corrections (11.9%), news items (28.1%), letters (34.2%) and editorial material (36.3%).

However, it is apparent that these patterns do not apply to the social media metrics. The probability to appear on Twitter is the highest for news items. While the overall percentage of documents with at least one tweet is at 21.5%, the probability for a news item to be tweeted is almost twice as high (42.3%). A more detailed analysis on journal level revealed that 23.4% (27.5% if merged with *BMJ-British Medical Journal*) of all 4,880 publications declared as news items were published in the *British Medical Journals*, 11.3% in *Veterinary Record*, 7.2% in *Plant Disease* and 4.7% in the *Canadian Medical Association Journal*. The fact that BMJ receives 62.4% (70.6%) of all tweets to news items and has a Twitter account (@bmj_latest) with more than 15,200 tweets and 127,000 followers raises another issue of the role of journal Twitter policies on Twitter impact of its papers and the general easiness to alter social media metrics. Reviews (36.2%) show the second highest Twitter coverage, followed by editorials (27.3%) and, finally, articles (21%). Although much lower, similar patterns can be observed for Google+ (news items 3.9%, reviews 1.3%, editorials 1.3%), Facebook (reviews 8.8%, news items 7.4%, editorials 7.4%) and blogs (reviews 2.5%, editorials 2.3%, news 2.2%), while mainstream media focus more on editorial material (0.8%) and articles (0.7%), albeit with much lower coverage. Mainstream media do not pick up book reviews or biographical items. Contrary to all other metrics, blogs are more likely to pick up corrections, which might be an indicator for them to discuss errors or retracted papers. Upon closer investigation, we found that the average number of mentions of retraction notices (338 retraction notices were identified by searching for “retraction”: containing “*retraction*” or papers beginning with “*retraction*” in the paper title. This includes all retraction notices published in 2012.) and retracted papers (164 retracted papers were identified by searching for “retracted”: containing “*retracted*” or beginning with “*retracted*” in the paper title. This includes all papers from 2012 which have been identified as retracted in WoS until mid-2013) in blogs is particularly high, a finding that might be due to blogs like Retraction Watch (http://retractionwatch.com/), which cover most retracted papers. Compared to an average of 0.03 blog posts for all documents (density), retraction notices are on average mentioned in blogs 0.35 times, and retracted papers 0.25 times. Mainstream media discuss retracted papers more often (0.03) than all documents on average (0.01) but do not (formally) cite the retraction notice. On Twitter, both retraction notices (1.10) and retracted papers (1.12) are mentioned more frequently than all papers on average (0.78).

Interestingly, news items and editorial material actually exhibit the highest density of tweets, even exceeding citation density. According to WoS ‘editorial material’ is defined as “an article that gives the opinions of a person, group, or organization. This includes editorials, interviews, commentary, and discussions between individual, post-paper discussions, round table symposia, and clinical conferences”. It is however important to remark that the definition of ‘editorial material’ in the WoS is not free of limitations, and it has been discussed that some publications labeled with this document type could be also considered as more regular articles [38]. In other words, these document types receive on average more tweets than citations. This fact supports the idea that document types that focus on topical subjects, debates and opinions, which are probably presented in simpler and less technical language, are more likely to appear and become popular on Twitter.

Independent of the probability of a certain document type to be mentioned on various platforms, the intensity confirms the popularity of news items on Twitter. Each news item mentioned on Twitter is (re-)tweeted by 7.1 users on average. Although the probability of editorials to be tweeted is slightly lower than that of reviews, they manage to attract a larger audience, reflected by an average of 5.8 users per tweeted document. Reviews (3.8) and articles (3.5) receive almost the same attention once on Twitter. However, based on the density, which is influenced by the coverage, review articles (1.4) are tweeted twice as much as articles (0.7). This analysis also allows for exploring some of the other social media sources in more detail. For example, we can see a slightly higher intensity of blog mentions for articles, letters and editorial material. Mainstream media mentions mention letters, news items and editorial material more frequently. However, density scores are very low overall due to the large number of documents without any captured activity on these platforms. Regardless of the low uptake, the intensity reveals slight differences between Facebook, Google+, media and blogs, although low scores close to 1 indicate that in general, social and mainstream media metrics are binary. Mainstream media seem to occur twice as often for letters and Facebook posts twice as often for editorials. Google+ users seem to (re-)post slightly more articles, whereas blogs occur more than once for articles, letters and editorial material. Taking into account documents without any social media counts (density), hardly any signals can be observed with the exception of Facebook for reviews (0.16), editorial material (0.15) and news items (0.12).

**Fig 1:**
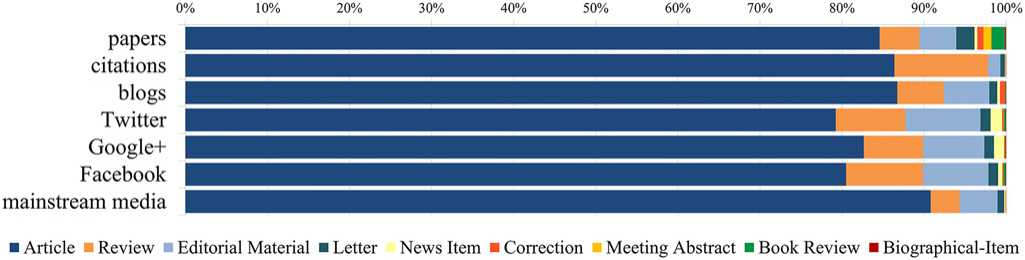
Percentage of counts referring to a particular document type. Percentage of papers, citations and social and mainstream media counts mentioning a particular WoS document type. The papers distribution serves as a reference of expected values if all documents were equally cited or mentioned.

The distribution of counts across document types (Fig. 1) (i.e. the percentage of citations and social media mentions referring to a certain document type) shows that citations are concentrated mainly on articles and review papers. However, blogs mention reviews less frequently (5.6% vs. 11.4%), editorials more often (5.6% vs. 1.5%) and corrections more often (0.6% vs. 0.1%). These results are similar but not identical to findings by Shema, Bar-Ilan and Thelwall [39], who analyzed the references in a sample of 391 blogs posts from the health category of Researchblogging.org and found that 71% of blog citations referred to articles, 14% to reviews, 3% to editorials, 2% to books, 2% to government and non-profit documents, 2% to proceedings and meeting abstracts, 2% to letters and 1% to news items. Mainstream media reference the largest share of articles (90.8%), followed by editorial material (4.6%) and reviews (3.5%), and Twitter with the lowest share of articles (79.2%) and highest share of editorials (9.1%) and news items (1.4%). This confirms findings shown in Table 2 and again underlines the importance of editorials and news on Twitter. Google+ and Facebook are comparable to the document type distribution on Twitter, with slightly higher shares of articles and fewer editorials as well as more reviews and fewer news items in the case of Facebook.

The results from Table 2 and Fig. 1 confirm that the document type influences the visibility of publications on social media. However, the document types which have a greater visibility on social media are not the same as those which have the highest citations. Most strikingly is the importance of news items and editorial material on Twitter, which supports the idea that newer, simpler and easy to understand publications attract the largest audiences on social media. The fact that review papers are also among the most covered across the different social media sources (with the exception of mainstream media, Table 2) suggests that publications that synthesize previous knowledge are attractive on Twitter.

### Correlations

This section focuses on the relationship between social media counts and bibliometric indicators. Table 3 presents Spearman correlations between these metrics based on all 1,339,279 documents (upper half of matrix) as well as those with the non-zero values of the respective social media events (lower half, values in italics). While the former values provide the general correlation between the new metrics and citations, the latter disregard documents with zero values for the social media metrics in a similar manner to the intensity shown in Table 2. Discipline-specific correlations on the level of the five LR fields can be found in S1 Table.

**Table 3:** Spearman correlation between variables.

	PG	NR	TI	AU	IN	CU	C	SC	B	T	F	G	M
**PG**	**1.000**	**0.622**	0.079	-0.006	0.116	0.131	0.194	0.159	-0.002	0.032	0.011	0.002	-0.010
**NR**		**1.000**	0.165	0.155	0.168	0.146	**0.416**	0.286	0.051	0.140	0.063	0.028	0.029
**TI**			**1.000**	**0.323**	0.135	0.038	0.142	0.117	-0.032	-0.004	-0.011	-0.027	-0.020
**AU**				**1.000**	**0.494**	0.252	0.281	0.225	0.030	0.089	0.049	0.010	0.033
**IN**					**1.000**	**0.560**	0.188	0.168	0.045	0.102	0.062	0.026	0.037
**CU**						**1.000**	0.155	0.166	0.043	0.061	0.039	0.023	0.035
**C**							**1.000**	**0.664**	0.124	0.194	0.097	0.065	0.083
**SC**								**1.000**	0.083	0.092	0.047	0.040	0.061
**B**	*-0*.*031*	*-0*.*022*	*-0*.*092*	*0*.*075*	*0*.*120*	*0*.*091*	*0*.*191*	*0*.*119*	**1.000**	0.194	0.180	0.196	0.279
**T**	*0*.*011*	*0*.*044*	*-0*.*093*	*0*.*011*	*0*.*084*	*0*.*056*	*0*.*148*	*0*.*065*		**1.000**	**0.320**	0.142	0.137
**F**	*-0*.*016*	*0*.*024*	*-0*.*042*	*0*.*065*	*0*.*084*	*0*.*049*	*0*.*167*	*0*.*079*			**1.000**	0.144	0.161
**G**	*-0*.*040*	*-0*.*005*	*-0*.*114*	*0*.*069*	*0*.*098*	*0*.*091*	*0*.*209*	*0*.*136*				**1.000**	0.179
**M**	*-0*.*092*	*-0*.*053*	*-0*.*090*	*0*.*118*	*0*.*135*	*0*.*127*	*0*.*199*	*0*.*127*					**1.000**

Medium correlations (≥±0.300) are highlighted in bold. Italic values indicate correlations based on papers with at least one of the respective events, i.e. blogs (n = 24,971), Twitter (n = 287,886), Facebook (n = 62,887), Google+ (n = 10,082) and mainstream media (n = 9,172) mentions

Based on all documents (including zero values, Table 3 upper half), the correlation analysis shows that among social media metrics, citations correlate the most with Twitter, although Spearman’s *ρ* is as low as 0.194 which confirms findings by Haustein et al. [28], Costas et al. [30] and others that tweets are not a good predictor of citation impact. It should be noted at this point that Mendeley, which is not included in this study, can be considered as the social media metric with the strongest correlation values with citations of around 0.5 [27,33,40]. As shown in Table 3, blogs follow Twitter with a Spearman correlation of 0.124 with citations. Citations show a positive correlation with the number of references (0.416), the collaboration indicators (particularly the number of authors: 0.281), and paper length (0.194); these findings have been documented in many other bibliometric studies (e.g., [41–47]).

Interestingly, social and mainstream media counts correlate the highest among each other—particularly Twitter and Facebook (0.320) and blogs and mainstream news (0.279)—and reveal only small correlations with indicators of collaboration and document characteristics. This indicates a similarity between the particular platforms in terms of which scientific papers are mentioned, and might also suggest that there are feedback effects with the social media echoing each other—an effect similar to what Bourdieu has called the *circular circulation of information* [48]. Low positive values can be found between Twitter and the number of references (0.140) as well as the number of collaborating institutions (0.102) of the paper. It is also interesting to note the negative correlations between the length of titles and all social media metrics, which increase when only non-zero publications are considered. Although the very low correlation values advise for caution in the interpretation, they might be an indication of the preference for short titles—which are perhaps simpler or more appealing—among these social media sources.

Low to moderate correlations with and among social media metrics are likely caused and influenced by the large amount of zero values, i.e. low coverage of documents on social media and news platforms. Mainstream media and Google+, which with 99% exhibit the highest shares of zero counts either due to actual low use or the inability to capture them, also show the lowest correlations with citations. On the other hand, with 22% non-zero values, Twitter exhibits the highest Spearman value with citations. Calculating Spearman’s *ρ* only for non-zero counts for each social media indicator clearly overestimates similarities with citation patterns but at the same time makes it independent of zero values. It is, however, important to note that based on this non-zero approach for each of the metrics (italic values in lower half of Table 3), the ranking changes and Google+ exhibits the highest correlations with citations (0.209; n = 10,082), followed by news (0.199; 9,172), blogs (0.191; 24,971) and Facebook (0.167; n = 62,887), while Twitter shows the lowest Spearman value (0.148; 287,886). It is also important to remark that the low correlation between citations and social media mentions, even when zero-values are excluded, reinforces once again the idea that citations and social media mentions are indeed different.

### Relationship among metrics and document characteristics

**Fig 2:**
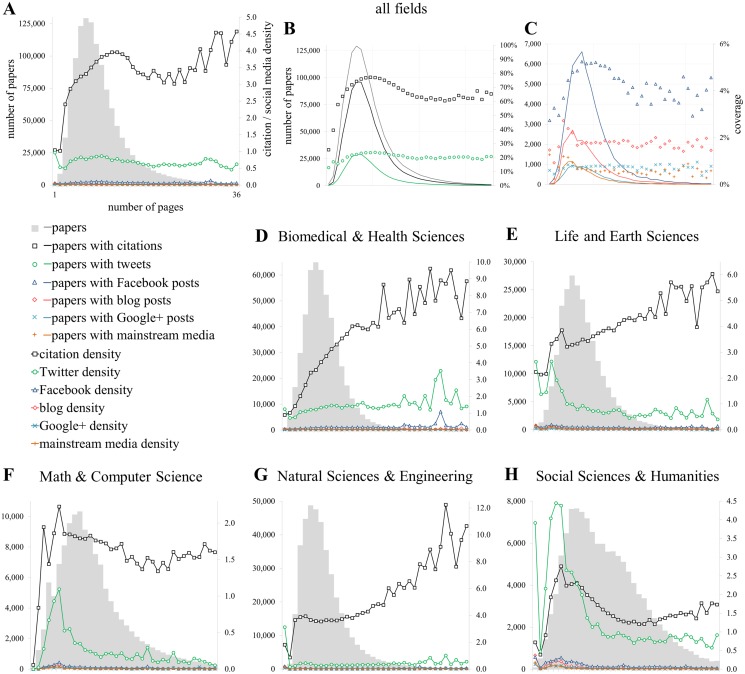
Relationship with the length of the publications [PG]. Proportion of publications of getting at least one metric (coverage; B, C) and citation and social media density (A, D-H) conditioned by the number of pages.

**Fig 3:**
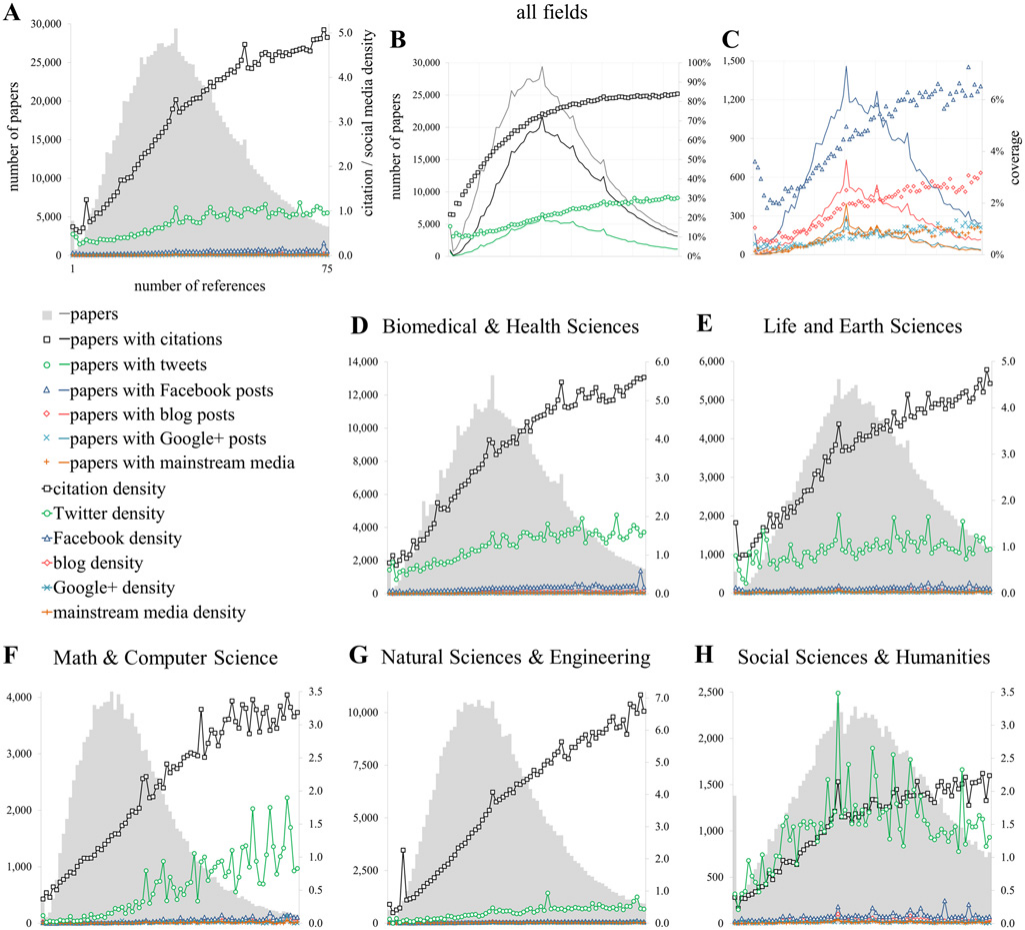
Relationship with the number of references [NR]. Proportion of publications of getting at least one metric (coverage; B, C) and citation and social media density (A, D-H) conditioned by the number of references (A, D-H).

**Fig 4:**
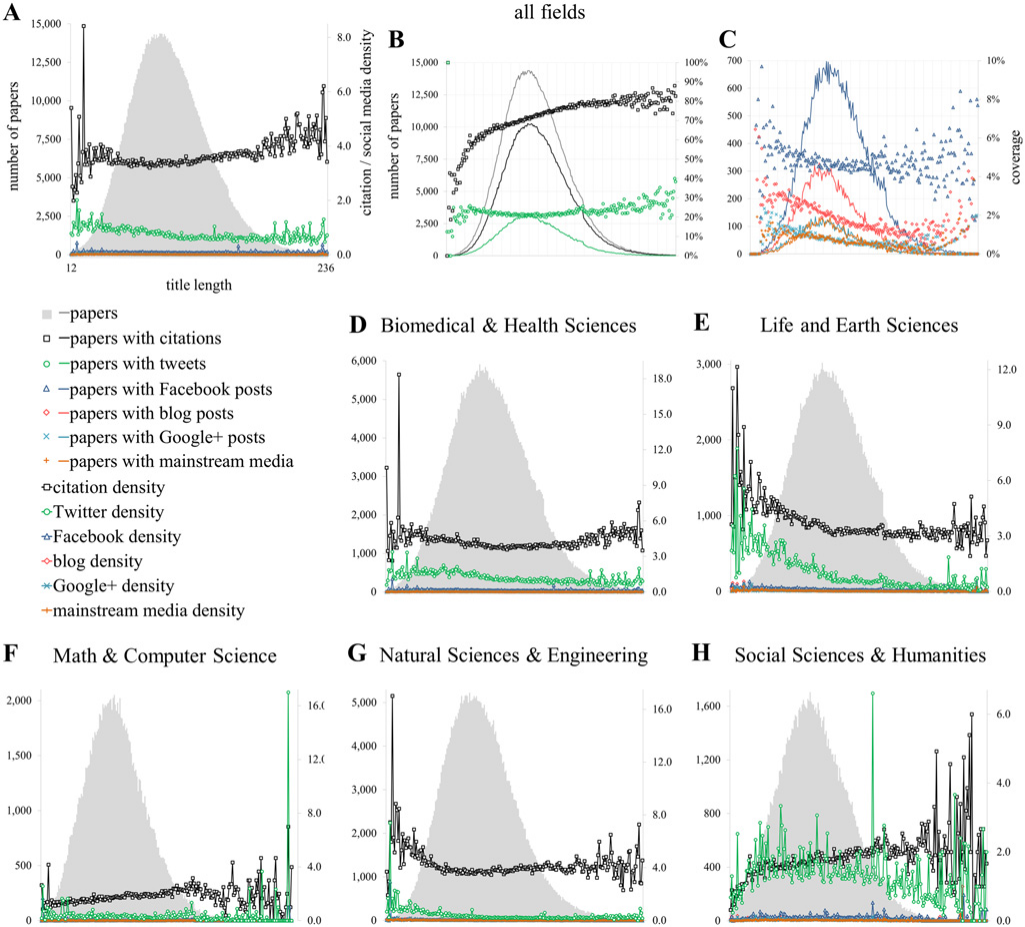
Relationship with the title length [TI]. Proportion of publications of getting at least one metric (coverage; B, C) and citation and social media density (A, D-H) conditioned by the number of characters in the title (A, D-H).

**Fig 5:**
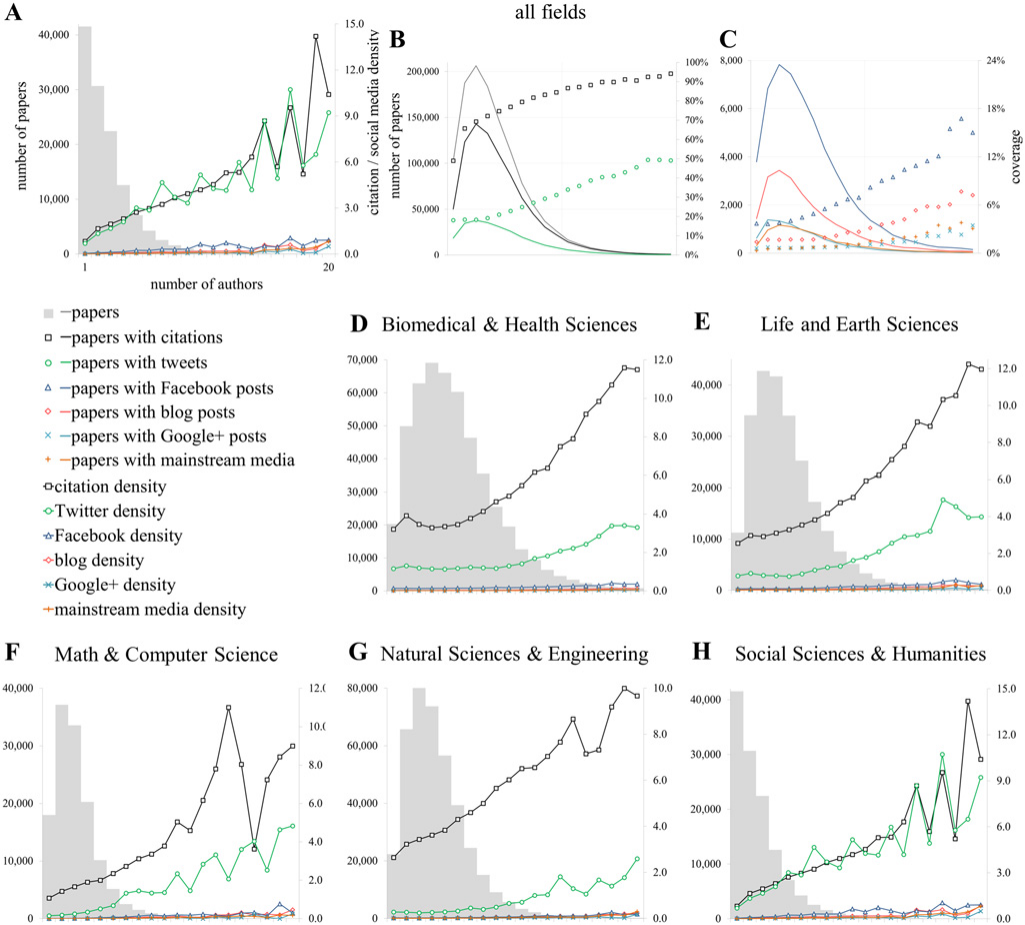
Relationship with the number of authors [AU]. Proportion of publications of getting at least one metric (coverage; B, C) and citation and social media density (A, D-H) conditioned by the number of authors (A, D-H).

To examine the relationship between the variables in more detail, simple regressions were made between document characteristics (PG, NR, TI) and collaboration indicators (AU, IN, CU), and averages of citations and social media metrics. As social media metrics are intended to capture the broader impact and/or predict the scientific impact of research, the regression analysis was limited to articles and reviews only, as the other document types are typically not peer-reviewed or considered as original contributions to knowledge. Regressions were carried out per LR field for the data shown in Figs. 2–5 as well as S2 and S3 Figs.

Two approaches were followed in order to perform this analysis. The first approach plots the average density values (i.e. the average of all observations, including zero-values) of the different metrics (i.e. C, B, T, F, G and M) conditioned by the different document and collaboration variables (i.e. PG, NR, TI, AU, IN and CU). In this analysis, we plot values with some minimum number of occurrences, as some rare cases (e.g. papers with extreme numbers of authors, pages, title length, etc.) tend to distort the graphs. These graphs are meant to exhibit the main patterns for citations and social media metrics, allowing for easy detection of the main differences and similarities. The second approach provides simple linear regression equations in S2 Table, which could not be included in the figures due to space limitations. Simple linear regression equations provide an easy interpretation of the relationships between the variables, and although in certain cases adjustments other than linear would have worked better, we focus on the linear approach because it simplifies the comparison among the different groups (e.g. the disciplines and the different metrics). It is important to mention that this linear regression analysis is based on the average of the different values conditioned by the characteristics of the papers (e.g. pages, references, etc.) and that it is a simplification of already summarized data. The interpretation of the data and figures mostly focuses on patterns for citations and Twitter, since the signals for the other social media and mainstream media are very low.

#### Document characteristics

Fig. 2 presents the relationship between the length of documents, citations and social media metrics. It is organized as follows: Fig. 2A presents the relationship between the density (i.e. average citation and social media rates including zero values) of citations and social media metrics as a function of the number of pages. The minimum and maximum values of pages (i.e. 1 and 36) are presented and are the same for Figs. 2B-2H. Figs. 2B (papers, citations and tweets) and 2C (Facebook, blogs, Google+ and mainstream media) shows the proportion of publications with at least one metric conditioned by the number of pages, while Figs. 2D-2H provides these values for the different LR fields. In the background of all graphs except B and C, the distribution of the number of publications by the number of pages is presented. The same structure is used for Figs. 3–5, S2 and S3 Figs.

Fig. 2A shows that the relationship between the number of pages and citations is positive and exhibits a kind of curvilinear relationship. Twitter mentions behave differently: the average density of tweets does not increase with the number of pages. S2 Table shows that the R^2^ of the relationship is very low (R^2^ = 0.250), and has a slightly negative slope. Due to values close to 0, the other sources (F, G, M) are hardly visible in Fig. 2A. According to their regression equations in S2 Table, the R^2^ values are quite small for all of them (<0.267) and the slopes tend to be slightly negative.

Fig. 2B shows that the chances of receiving at least one citation increases gradually from 25.6% (1 page papers) to 77% (11 pages papers), after which the percentage of papers with citations decreases again to reach an average citedness rate of 63% between 20 and 36 pages. Although much lower, the percentage of papers with at least one public Facebook post (Fig. 2C) shows a similar pattern, while the curves for Twitter (Fig. 2B), blogs, Google+ and mainstream media (Fig. 2C) are much flatter and do not show much fluctuation after papers with 6 pages or more. Based on the distribution of papers with at least one tweet, the length of papers seems only to have a small effect on the probability of a publication to be mentioned on Twitter. From a more critical perspective, it could also be argued that the length of a paper does not affect its probability of being tweeted because papers might not actually be read when tweeted, and that factors such as curious topics or funny titles increases Twitter counts [28,49].

Comparing disciplines (Figs. 2D-2H), papers in Mathematics and Computer Science and in the Social Sciences and Humanities are the largest in terms of pages (Table 1) while Biomedical and Health Sciences and Natural Sciences and Engineering are the shortest. In terms of density, it can be seen that, contrary to citations, popularity on Twitter goes down with increasing paper length in Life and Earth Sciences (Fig. 2E; *y = -0*.*0338x+1*.*5161* in S2 Table; *ρ = -0*.*014* in S1 Table), Math & Computer Science (Fig. 2F) and Social Sciences in Humanities (Fig. 2H). The Social Sciences and Humanities show a particular pattern, with tweet density matching or even exceeding citation rates, and very similar patterns for both metrics, i.e. a small peak for papers with 1 page, a steep increase to 6 pages and a gradual decrease for longer papers. If the average number of event counts is considered for papers with at least one event only (intensity), effects are even more pronounced (data not shown). Considering all document types (data not shown), papers of only one or two pages actually on average get more tweeted than cited, which is caused by the popularity of news items and editorial material, which are usually very short.

In Fig. 3, the relationship of metrics with the number of cited references per paper are explored. The top graphs show the density (Fig. 3A) and probability (Figs. 3B and 3C) of publications to get cited or mentioned on social media platforms as a function of their number of references. In the case of citations, the relationship with the number of references is strongly positive and the pattern is clear: the longer the reference list, the higher the chances of being cited. In the case of social media mentions, we can see how this pattern tends also to be positive with a bump in the Twitter curve for papers with between 4 and 20 references. It is also remarkable that for the papers with the shortest reference lists (~3 or less) the density of citations and Twitter mentions are very similar (Fig. 3A). On the whole, it is clear that for citations this relationship is positive, while for tweets we only see a slight positive trend. This can be corroborated in S2 Table, with y = 0.0622x for citations and y = 0.0111x for tweets; the increase is even smaller for the other social media mentions.

The highest R^2^ values can be observed in Biomedical and Health Sciences (Fig. 3G) across all metrics indicating the strongest positive linear effects compared to number of pages and characters in the title. Mathematics and Computer Sciences and Natural Sciences and Engineering also present high R^2^ values for blogs, tweets and Facebook posts. It should be noted that, while often small, regression lines across all metrics and all fields are positive for the number of references.

Fig. 4 explores the relationship between the different metrics and the length of the titles of scientific publications. The titles of articles and their length are related to how the authors try to capture the attention of their potential readers [50] and they can also serve as a rough estimator of the complexity (e.g. shorter titles tend to be linked to applied research while longer ones to be linked to basic research [51]) and the topic of the papers. We could argue that shorter titles might refer to more applied topics which might be more attractive to users on social media platforms. Fig. 4A shows that the number of characters in titles is normally distributed and Fig. 4B indicates that the probability of publications getting cited at least once also increases with the length of the titles, although in a curvilinear fashion. In the case of social media metrics, Twitter and Facebook counts behave similarly with higher counts for very short titles of less than 30 characters. For titles between 40 and 150 characters, which represent the vast majority of all papers, no effect of the length can be observed regarding a paper’s visibility on Twitter and Facebook.

The density of citation and social media impact as a function of title length (Fig. 4A) shows a different pattern. The expected average number of citations tends to increase with the length of the titles, with an average increase of around 0.0046 citations per extra character. However, for Twitter mentions we see the opposite: an increase in title length has a slightly negative effect on the number of tweets. Despite the fact that activity on Google+, mainstream media and blogs is very low, the regression slopes are negative (S2 Table).

However, this relationship varies across disciplines for citations as well as social media metrics. For instance, in Biomedical and Health Sciences (Fig. 4D), Life and Earth Sciences (4E) and Natural Sciences and Engineering (4G), the relationship between citations and title length is negative (S2 Table), while for Mathematics and Computer Science (4F) and the Social Sciences and Humanities (4H) it is positive—although only slightly. It is also remarkable that for all fields except Twitter in Mathematics and Computer Science, the slopes of the relationship between titles and social media metrics are systematically negative, although extremely small in all cases.

#### Collaboration

In this section we focus on citation and social media metrics regarding the level of collaboration. Given the causality (and correlations) between the number of authors, institutions and countries (cf. Table 3) we focus on one of these indicators, namely the number of authors, as the micro level of collaboration. However, results of these relationships for institutions and countries are provided in the supplementary material (S2 and S3 Figs.).

Fig. 5 provides the relationship between the different average values of the metrics and the number of authors. Fig. 5A shows that the average impact of publications systematically increases with the number of authors per paper, and this relationship is observed in each of the fields (Figs. 5D-5H). Social media metrics have the same pattern: the higher the number of authors, the higher the scores obtained (Figs. 5B and 5C). Based on the slope values of the linear regression (S2 Table), publications obtain on average almost 0.49 citations for each additional author, with an increase of 0.13 tweets and 0.01 Facebook posts on average. S2 Table and Figs. 5D and 5H show that this relationship can be observed in all fields. For all domains but the Social Sciences and Humanities, the effect of the number of authors is higher for citations than for tweets. In the Social Sciences and Humanities, citations and Twitter curves superpose each other almost perfectly, which is likely due to the low citation density of these disciplines as well as the relatively high Twitter activity. S2 and S3 Figs. provide similar results for the number of institutions and countries.

On the whole, Fig. 5 provides clear evidence that the relationship between the number of authors and citation and social media metrics—especially Twitter—is positive. The relationship between citations and number of authors has been well documented before [42,52–54], and is not caused by authors’ self-citations [47]. However, the effect of “self-mentions” in social media on these trends has yet to be assessed.

## Conclusions

Grouped under the umbrella term of “altmetrics”, a number of metrics based on social media platforms have recently been introduced as potential indicators of research impact, which could be “alternatives” to citations (as they take less time accumulate) or could perhaps indicate the broader impact of research on society [22]. These social media metrics are quite heterogeneous and refer to mentions of scientific articles on microblogging platforms such Twitter and Weibo, posts on social network sites such as Facebook and Google+, saves on social reference managers Mendeley and CiteULike, reviews on F1000Prime, Publons and PubPeer, as well as mentions in scholarly blogs and news and mainstream media. Thus, they encompass various types of usage, levels of engagement, user groups and audiences, are affected by different user uptake, and vary in terms of data availability. This heterogeneity affects their analysis, and complicates the construction of a proper interpretative framework for these indicators [25,27,28,30].

In order to better understand the factors affecting the social media metrics scores, this paper analysed their relationship with document properties and collaborative patterns, and compared them with what is known for citations. This comparison was based on 1.3 million documents published in journals indexed by the Web of Science in 2012 and corresponding data from Altmetric.com. Five social media metrics were considered: tweets, Facebook and Google+ posts, as well as mentions in scholarly blogs and news outlets.

Our results show that globally the presence of social media metrics across scientific publications is still quite low, with Twitter being the main platform despite the fact that only 22% of all publications considered in this study received at least one tweet. The prevalence of documents on social media is also determined by their type: coverage, density and intensity of Twitter mentions of editorial material and news items are much higher than that of articles, which is contrary to what is obtained for citations. This might be due to their shortness and simplicity, or to the fact that these news items generally present the results of chosen studies, which have often been selected because of their general interest to the scientific community or society in general. Along these lines, blogs have a higher than expected coverage of ‘corrections’*—*which might be due to blogs such as *Retraction Watch—*while mainstream media have an equivalent coverage of articles and editorials. Finally, the coverage of reviews is among the highest for several of the indicators (i.e. citations, blogs, Facebook), thus reinforcing the attractiveness of this particular document type, probably caused by their property of ‘synthesis’ of previous knowledge, which is also known for citations. Worth mentioning here are the differences across domains: Twitter and other social media are more frequent for the Social Sciences and Humanities and the Biomedical and Health Sciences and hardly present among Mathematics and Computer Science and Natural Sciences and Engineering. This suggests, as proposed by Haustein et al. [28] and Costas et al. [30] that social media metrics tend to focus more on social and health-related topics while other, more technical, mathematical or physical/chemistry topics, are less attractive for users of these platforms. Regarding certain bibliographic characteristics (PG, NR, TI, AU, IN, CU), tweets and citations follow a very similar behaviour in Social Sciences and Humanities. This finding, coupled with the fact that the domain has the highest correlation between the two indicators, suggests that citations and tweets are more related in the Social Sciences and Humanities than in other fields. The reason for and meaning behind this relation still needs to be further studied by exploring the motivation behind tweeting. Interestingly it is in the same domain where citer motivations are most controversial and citations as impact indicators most disputed.

Our results also confirmed the weak relationship between citations and social media metrics [25,28,30], with the highest correlation found between citations and Twitter and blogs. The correlation between Twitter and Facebook mentions, and to a lesser extent between blogs and mainstream media, can be highlighted, supporting the idea that these social media metrics are indicators of different types of visibility [30], despite the fact that there is a certain circularity on the diffusion of information [48] where, for example, documents found on Twitter are also more likely to be found on Facebook.

While it is well known that the number of pages and references, length of the titles, and number of authors, institutions and countries affect citations positively, this relationship is not as clear for social media indicators. More specifically, our results show that the length of titles and papers has, to a certain extent, a negative effect on social media counts, thus suggesting that the well-known brevity of social media echoes in the scientific papers it diffuses. This finding is also reinforced by the fact that shorter document types such as editorial material, news items and letters obtain higher visibility on these platforms—a pattern quite contrary to what is known for citations. Other aspects such as the number of references or the number of collaborators of papers do have a positive relationship with the various social media counts, although with a much lower effect compared to citations.

On the whole, our results confirm that citations and social media metrics are essentially different. Not only are indicators obtained at the level of papers weakly correlated, but factors that typically affect citations rates do not seem to affect social media counts in a similar manner. Therefore, social media metrics cannot actually be seen as alternatives to citations, but at most as complements to other type of indicators. This complementarity, however, still needs much research to be better understood. We hope that this paper, by studying the relationship between social media, citations, as well as other factors that typically influence citation rates, contributed to this understanding.

## Supporting Information

S1 Fig Presence of DOIs per journal per LR field. Percentage of journals with a certain percentage of documents with a DOI.
(TIF)

S2 Fig Relationship with the number of institutions [IN]. Proportion of publications of getting at least one metric (coverage; B, C) and citation and social media density (A, D-H) conditioned by the number of institutions (A, D-H).
(TIF)

S3 Fig Relationship with the number of countries [CU]. Proportion of publications of getting at least one metric (coverage; B, C) and citation and social media density (A, D-H) conditioned by the number of countries (A, D-H).
(TIF)

S1 Table Spearman correlation between variables per LR field. Based on all documents per field; medium correlations (≥±0.300) are highlighted in bold.
(DOCX)

S2 Table Linear regression equations per variables and LR fields. Equations for linear regressions based on citation (C) and social media density (B, T, F, G, M) shown in Figs. 2–5 and S2 and S3 Figs. Positive and negative slopes with β≥±0.001as well as coefficients of determination R^2^≥0.300 are highlighted in bold.
(DOCX)

## References

[1] Cole F, Eales N. The history of comparative anatomy. Part I: A statistical analysis of the literature. Science Progress. 1917;11:578–96.

[2] Gross PL, Gross EM. College libraries and chemical education. Science. 1927;66(1713):385–9. 17782476 10.1126/science.66.1713.385

[3] Lotka AJ (1926) The frequency distribution of scientific productivity. Journal of Washington Academy Sciences. 1926;16:317–23.

[4] Zipf GK. Human behavior and the principle of least effort. Cambridge: Addison-Wesley Press; 1949.

[5] Bradford SC. Sources of information on specific subjects. Engineering. 1934;137:85–6.

[6] Garfield E. Science Citation Index—A new dimension in indexing. Science. 1964;144(3619):649–54. 17806988 10.1126/science.144.3619.649

[7] Clark BL. Multiple authorship trends in scientific papers. Science. 1964;143(3608):822–4. 14088083 10.1126/science.143.3608.822

[8] Falagas ME, Zarkali A, Karageorgopoulos DE, Bardakas V, Mavros MN. The Impact of Article Length on the Number of Future Citations: A Bibliometric Analysis of General Medicine Journals. PLOS ONE. 2013;8(2):e49476 10.1371/journal.pone.0049476 23405060 PMC3566179

[9] Lovaglia MJ. Predicting citations to journal articles: The ideal number of references. The American Sociologist. 1991;22(1):49–64

[10] Cole JR, Cole S. Social Stratification in Science. Chicago: University of Chicago Press; 1973.

[11] Cronin B. The citation process The role and significance of citations in scientific communication. London: Taylor Graham; 1984.

[12] Merton RK. The sociology of science: Theoretical and empirical investigations. Chicago: University of Chicago Press; 1973.

[13] Nicolaisen J. Citation Analysis. Annual Review of Information Science and Technology. 2007;41:609–41.

[14] Wouters P. The Citation Culture. Amsterdam: University of Amsterdam; 1999.

[15] Zuckerman H. Citation analysis and the complex problem of intellectual influence. Scientometrics. 1987;12(5–6):329–38.

[16] Edge D. Quantitative Measures of Communication in Science: A Critical Review. History of Science. 1979;17:102–34 11610633 10.1177/007327537901700202

[17] MacRoberts MH, MacRoberts BR. Another test of the normative theory of citing. Journal of the American Society for Information Science. 1987;38:305–6.

[18] MacRoberts MH, MacRoberts BR. Citation analysis and the science policy arena. Trends in Biochemical Science. 1989;14(1):8–12.

[19] Bornmann L, Daniel HD. What do citation counts measure? A review of studies on citing behavior. Journal of Documentation. 2008;64(1):45–80

[20] Neylon C, Wu S. Article–level metrics and the evolution of scientific impact. PLOS Biology. 2009;7(11):e1000242 10.1371/journal.pbio.1000242 19918558 PMC2768794

[21] Priem J, Hemminger BM. Scientometrics 2.0: Toward new metrics of scholarly impact on the social Web. First Monday. 2010;15(7). Available from: http://firstmonday.org/article/viewArticle/2874/257022.

[22] Prie J, Taraborelli D, Groth P, & Neylon C. Altmetrics: a manifesto; 2010. Available from: http://altmetrics.org/manifesto/

[23] Rousseau R, Ye FY (2013) A multi-metric approach for research evaluation. Chinese Science Bulletin. 2013;58(26):10–2.

[24] Haustein S, Bowman TD, Holmberg K, Tsou A, Sugimoto CR, Larivière V. Automated Twitter accounts in scholarly communication and their effects on tweets as impact indicators. Journal of the Association for Information Science and Technology. Forthcoming 2015 Available from: http://arxiv.org/abs/1410.4139

[25] Thelwall M, Haustein S, Larivière V, Sugimoto CR. Do altmetrics work? Twitter and ten other candidates. PLoS ONE. 2013;8(5):e64841 10.1371/journal.pone.0064841 23724101 PMC3665624

[26] Wouters P, Costas R. Users, narcissism and control—tracking the impact of scholarly publications in the 21 st century. Stichting: SURFfoundation; 2012. Available from: http://www.surf.nl/binaries/content/assets/surf/en/knowledgebase/2011/Users+narcissism+and+control.pdf

[27] Zahedi Z, Costas R, Wouters P. How well developed are altmetrics? A cross-disciplinary analysis of the presence of “alternative metrics” in scientific publications. Scientometrics. 2014;101(2):1491–513.

[28] Haustein S, Peters I, Sugimoto CR, Thelwall M, Larivière V. Tweeting Biomedicine: An Analysis of Tweets and Citations in the Biomedical Literature. Journal of the Association for Information Science and Technology. 2014;65(4):656–69.

[29] Robinson-García N, Torres-Salinas D, Zahedi Z, Costas R. New data, new possibilities: exploring the insides of Altmetric.com. El Profesional de La Informacion. 2014;23(4):359–66.

[30] Costas R, Zahedi Z, Wouters P. Do “altmetrics” correlate with citations? Extensive comparison of altmetric indicators with citations from a multidisciplinary perspective. Journal of the Association for Information Science and Technology. Forthcoming 2015 Available from: http://arxiv.org/abs/1401.4321

[31] Haustein S, Larivière V, Thelwall M, Amyot D, Peters I. Tweets vs. Mendeley readers: How do these two social media metrics differ? It—Information Technology. 2014;56(5):207–15.

[32] Li X, Thelwall M. F1000, Mendeley and Traditional Bibliometric Indicators In: Archambault E, Gingras Y, Larivière V, editors. 17th International Conference on Science and Technology Indicators. Montreal: Science-Metrix and OST; 2012 Sep 5–8; Vol. 2, p. 541–51.

[33] Mohammadi E, Thelwall M, Haustein S, Larivière V. Who Reads Research Articles ? An Altmetrics Analysis of Mendeley User Categories. Journal of the Association for Information Science and Technology. Forthcoming 2015 Available from: http://www.scit.wlv.ac.uk/~cm1993/papers/WhoReadsResearchArticlesPreprint.pdf

[34] Waltman L, Costas R. F1000 Recommendations as a Potential New Data Source for Research Evaluation: A Comparison With Citations. Journal of the Association for Information Science and Technology. 2014;65(3):433–45. 25346934

[35] Neylon C. Altmetrics: What are they good for? PLOS Opens blog post from 3 October 2014. Available from: http://blogs.plos.org/opens/2014/10/03/altmetrics-what-are-they-good-for/

[36] Harzing AW. Document categories in the ISI Web of Knowledge: Misunderstanding the Social Sciences? Scientometrics. 2012;94(1):23–34.

[37] Sigogneau A. An analysis of document types published in journals related to physics: Proceeding papers recorded in the Science Citation Index database. Scientometrics. 2000;47(3):589–604.

[38] Van Leeuwen T, Costas R, Calero-Medina C, Visser M. The role of editorial material in bibliometric research performance assessments. Scientometrics. 2012;95(2):817–28.

[39] Shema H, Bar-Ilan J, Thelwall M. How Is Research Blogged? A Content Analysis Approach. Journal of the Association for Information Science and Technology. Forthcoming 2015.

[40] Li X, Thelwall M, Giustini D. Validating online reference managers for scholarly impact measurement. Scientometrics. 2011;91(2):461–71. 10.1152/physrev.00011.2010 21527731

[41] Biglu MH. The influence of references per paper in the SCI to Impact Factors and the Matthew Effect. Scientometrics. 2008;74(3):453–70.

[42] Costas R, Van Bochove C. On the Relationship between Author Collaboration and Impact of Scientific Publications In: Archambault E, Gingras Y, Larivière V, editors. 17th International Conference on Science and Technology Indicators. Montreal: Science-Metrix and OST; 2012 Sep 5–8; Vol. 2, p. 447–8.

[43] Costas R, Van Leeuwen TN. Referencing Patterns of Individual Researchers: Do Top Scientists Rely on More Extensive Information Sources? Journal of the American Society for Information Science and Technology. 2012;63(12):2433–50.

[44] Hsu J, Huang D. Correlation between impact and collaboration. Scientometrics. 2011;86(2):317–24.

[45] Onodera N, Yoshikane F. Factors Affecting Citation Rates of Research Articles. Journal of the Association for Information Science and Technology. Forthcoming 2015.

[46] Webster GD, Jonason PK, Orozco T. Hot Topics and Popular Papers in Evolutionary Psychology: Analyses of Title Words and Citation Counts in Evolution and Human Behavior, 1979–2008. Evolutionary Psychology. 2009;7(3):348–62.

[47] Larivière V, Sugimoto CR, Tsou A, Gingras Y. Team size matters: Collaboration and scientific impact since 1900. Journal of the American Society for Information Science and Technology. Forthcoming 2015. Available from: http://arxiv.org/abs/1410.8544 10.1002/asi.22796 24729747 PMC3981099

[48] Bourdieu P. On Television. New York: The New Press; 1999.

[49] Sud P, Thelwall M. Evaluating altmetrics. Scientometrics. 2014;98(2):1131–43.

[50] Buter RK, van Raan AFJ. Non-alphanumeric characters in titles of scientific publications: An analysis of their occurrence and correlation with citation impact. Journal of Informetrics. 2011;5(4):60–17.

[51] Lewison G, Hartley J. What’s in a title? Number of words and the presence of colons. Scientometrics. 2005;63(2):341–56.

[52] Bordons M, Zulueta MA, Barrigon S. Scientific Activity of the Most Productive Spanish Research Teams in Pharmacology and Pharmacy During the Period 1986–1993 as Covered by the Science Citation Index (Sci). Medicina Clinica. 1998;111(13):489–95. 9859065

[53] Glänzel W, Schubert A. Double effort = Double impact? A critical view at international co-authorship in chemistry. Scientometrics. 2001;50(2):199–214.

[54] Herbertz H. Does it pay to cooperate? A bibliometric case study in molecular biology. Scientometrics. 1995;33(1):117–22.

