# Correction: Characterizing Social Media Metrics of Scholarly Papers: The Effect of Document Properties and Collaboration Patterns

**DOI:** 10.1371/journal.pone.0127830

**Published:** 2015-05-08

**Authors:** Stefanie Haustein, Rodrigo Costas, Vincent Larivière

In Fig 5, Panel H is a duplicate of Panel A. Please see the corrected Fig 5 here.

**Fig 5:**
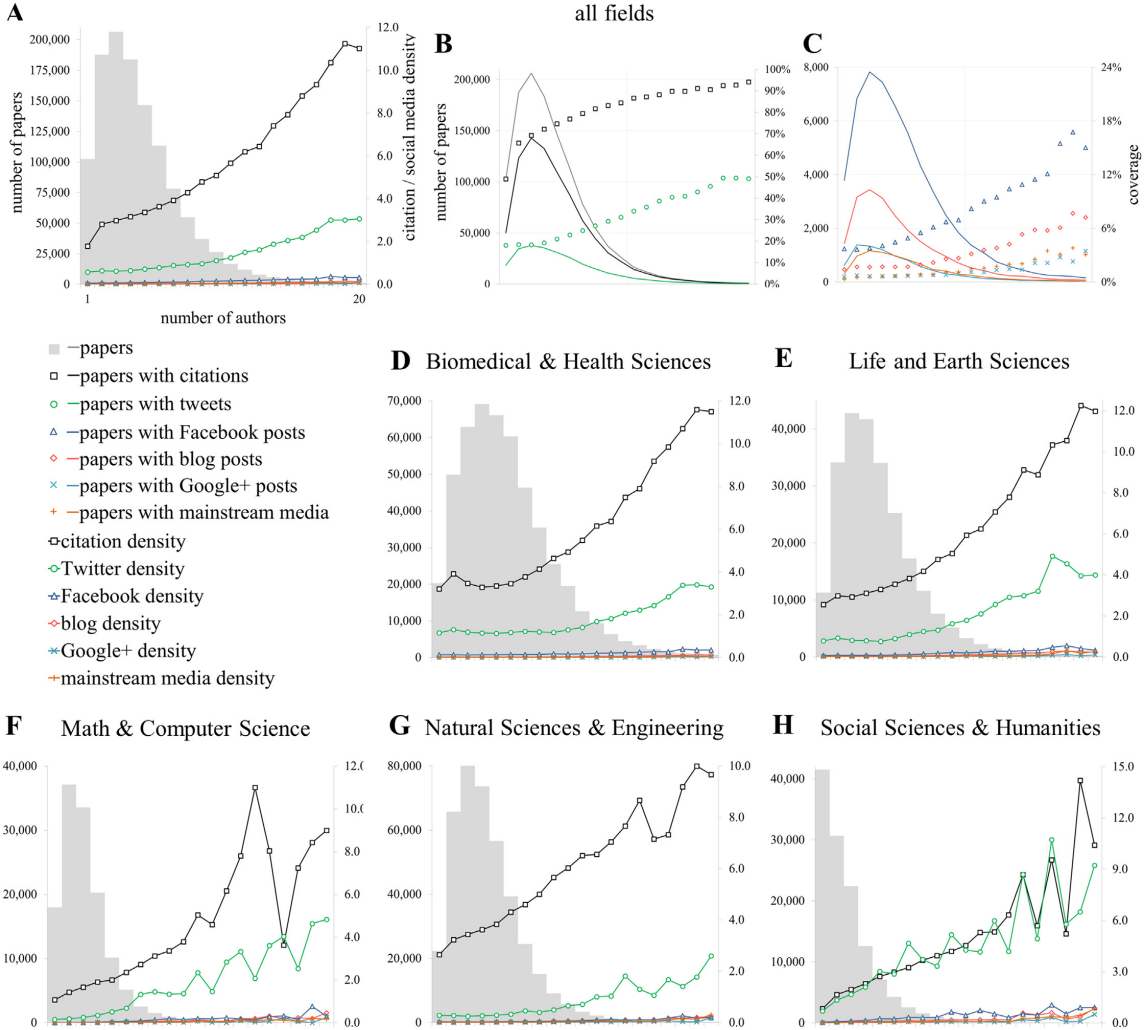
Relationship with the number of authors [AU]. Proportion of publications of getting at least one metric (coverage; B, C) and citation and social media density (A, D-H) conditioned by the number of authors (A, D-H).
