# Supplementary figures and images for: Characterizing Social Media Metrics of Scholarly Papers: The Effect of Document Properties and Collaboration Patterns
Source: PLoS One. 2015 Mar 17;10(3):e0120495. doi: 10.1371/journal.pone.0120495 (PMC4363625; doi:10.1371/journal.pone.0120495)

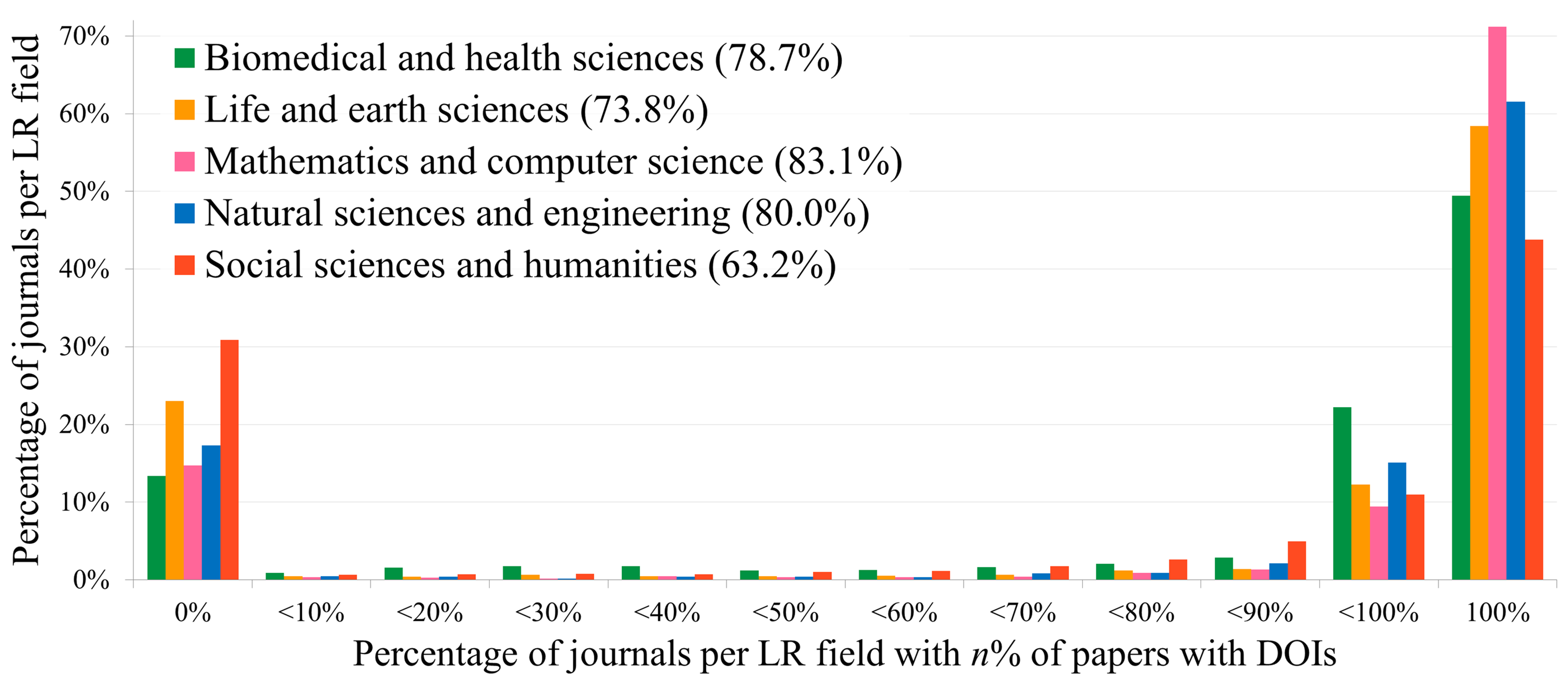

Supplement: S1 Fig — Percentage of journals with a certain percentage of documents with a DOI. (TIF) [file pone.0120495.s001.tif]

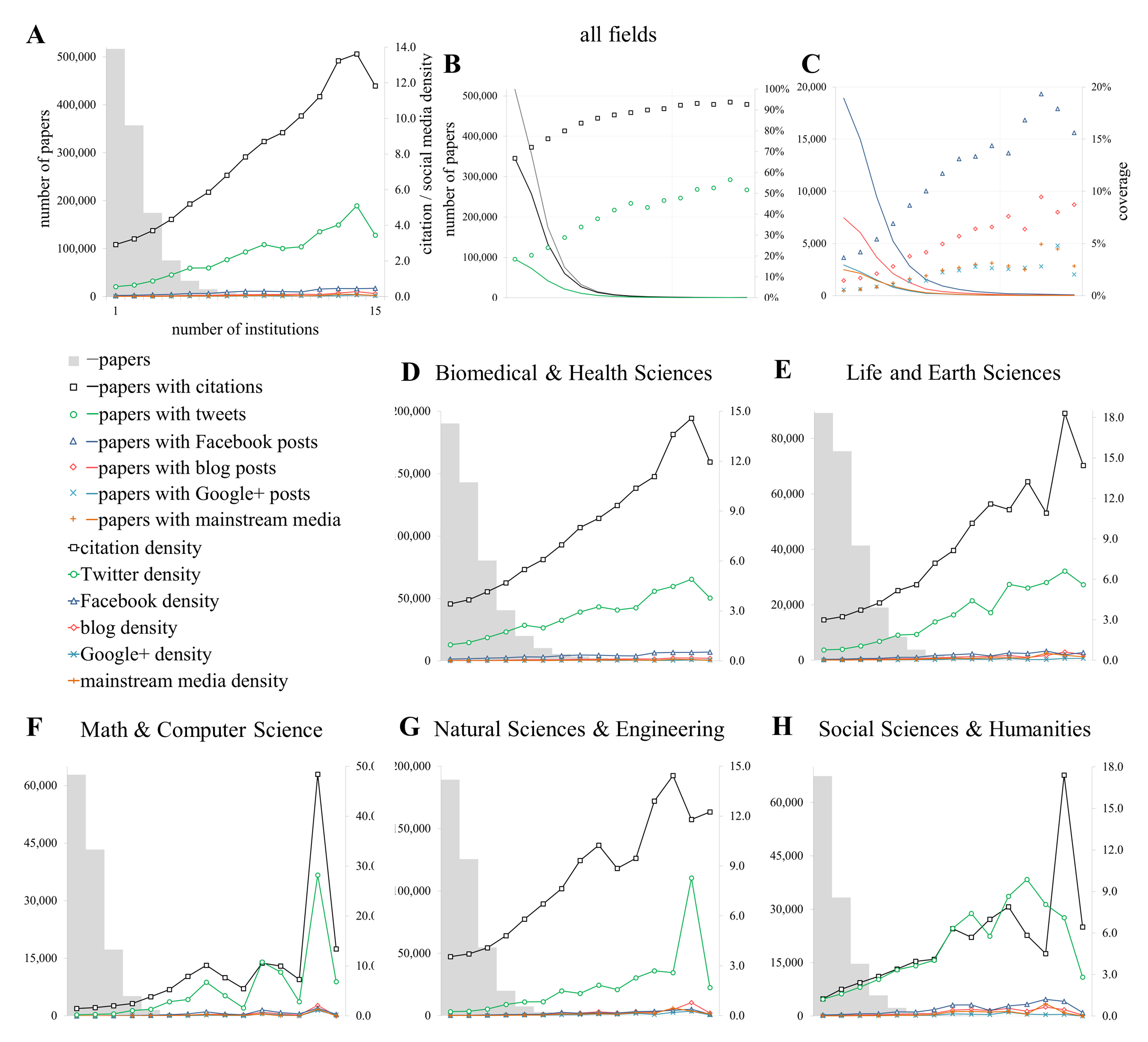

Supplement: S2 Fig — Proportion of publications of getting at least one metric (coverage; B, C) and citation and social media density (A, D-H) conditioned by the number of institutions (A, D-H). (TIF) [file pone.0120495.s002.tif]

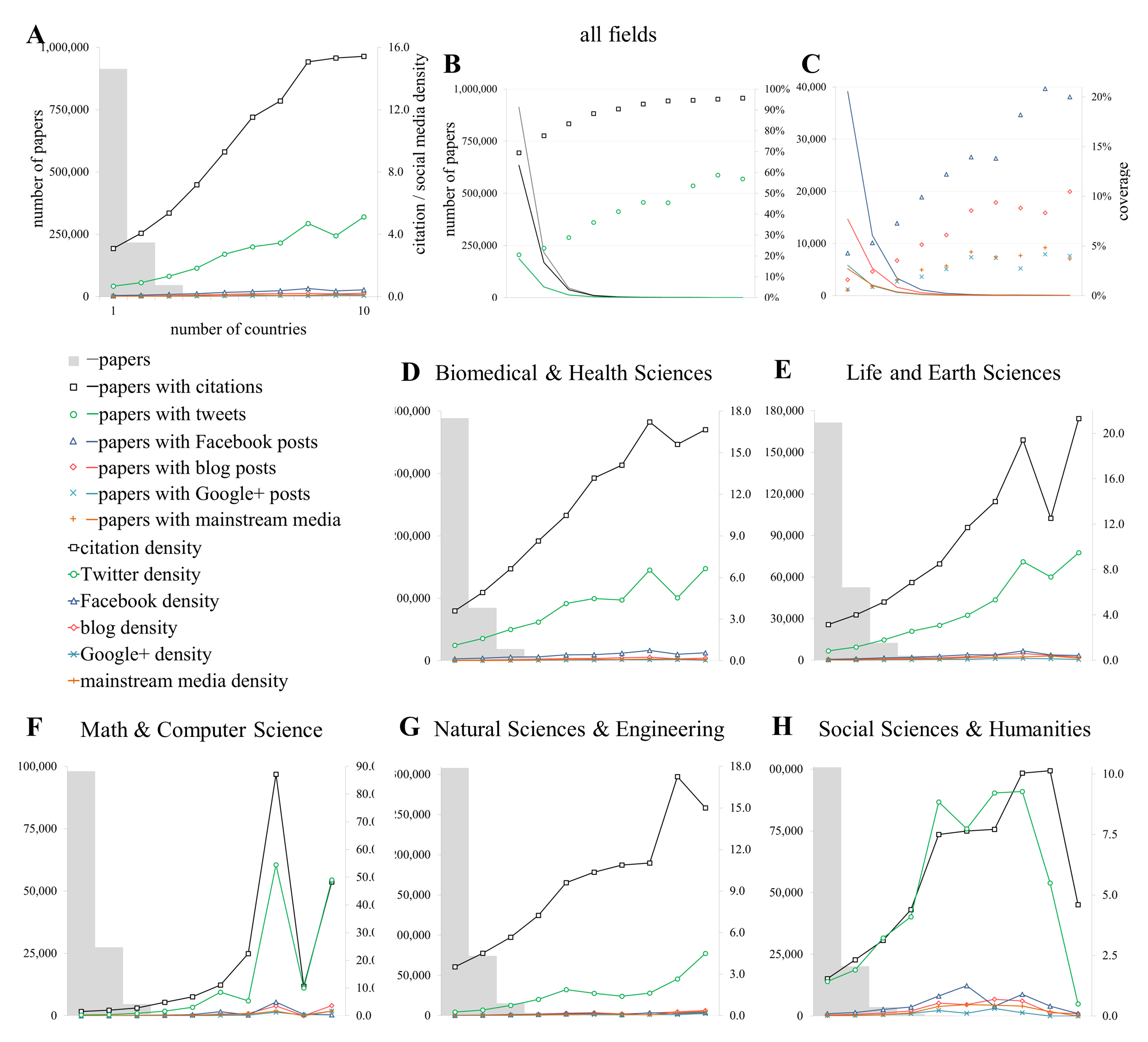

Supplement: S3 Fig — Proportion of publications of getting at least one metric (coverage; B, C) and citation and social media density (A, D-H) conditioned by the number of countries (A, D-H). (TIF) [file pone.0120495.s003.tif]
