# Supplementary material for: Characterizing Social Media Metrics of Scholarly Papers: The Effect of Document Properties and Collaboration Patterns
Source: PLoS One. 2015 Mar 17;10(3):e0120495. doi: 10.1371/journal.pone.0120495 (PMC4363625; doi:10.1371/journal.pone.0120495)
Supplement: S1 Table — Based on all documents per field; medium correlations (≥±0.300) are highlighted in bold. (DOCX) [file pone.0120495.s004.docx]

**S1 Table. Spearman correlation between variables per LR field.** Based on all documents per field; medium correlations (≥±0.300) are highlighted in bold.

| **LR field** |  |  | **PG** | **NR** | **TI** | **AU** | **IN** | **CU** | **C** | **SC** | **B** | **T** | **F** | **G** | **M** |
| --- | --- | --- | --- | --- | --- | --- | --- | --- | --- | --- | --- | --- | --- | --- | --- |
| Biomedical and health sciences |  | **PG** |  | **0.771** | 0.202 | 0.238 | 0.210 | 0.167 | **0.424** | **0.313** | 0.034 | 0.114 | 0.057 | 0.014 | 0.016 |
|  |  | **NR** | **0.771** |  | 0.124 | 0.167 | 0.176 | 0.155 | **0.465** | **0.314** | 0.050 | 0.138 | 0.074 | 0.028 | 0.031 |
|  |  | **TI** | 0.202 | 0.124 |  | **0.327** | 0.155 | 0.072 | 0.116 | 0.116 | -0.037 | -0.029 | -0.027 | -0.034 | -0.024 |
|  |  | **AU** | 0.238 | 0.167 | **0.327** |  | **0.483** | 0.264 | 0.248 | 0.228 | 0.021 | 0.048 | 0.029 | -0.002 | 0.029 |
|  |  | **IN** | 0.210 | 0.176 | 0.155 | **0.483** |  | **0.519** | 0.200 | 0.193 | 0.048 | 0.106 | 0.066 | 0.022 | 0.041 |
|  |  | **CU** | 0.167 | 0.155 | 0.072 | 0.264 | **0.519** |  | 0.175 | 0.187 | 0.048 | 0.082 | 0.054 | 0.023 | 0.038 |
|  |  | **C** | **0.424** | **0.465** | 0.116 | 0.248 | 0.200 | 0.175 |  | **0.630** | 0.129 | 0.211 | 0.115 | 0.072 | 0.087 |
|  |  | **SC** | **0.313** | **0.314** | 0.116 | 0.228 | 0.193 | 0.187 | **0.630** |  | 0.091 | 0.119 | 0.067 | 0.046 | 0.067 |
|  |  | **B** | 0.034 | 0.050 | -0.037 | 0.021 | 0.048 | 0.048 | 0.129 | 0.091 |  | 0.204 | 0.199 | 0.215 | 0.291 |
|  |  | **T** | 0.114 | 0.138 | -0.029 | 0.048 | 0.106 | 0.082 | 0.211 | 0.119 | 0.204 |  | **0.342** | 0.147 | 0.140 |
|  |  | **F** | 0.057 | 0.074 | -0.027 | 0.029 | 0.066 | 0.054 | 0.115 | 0.067 | 0.199 | **0.342** |  | 0.149 | 0.176 |
|  |  | **G** | 0.014 | 0.028 | -0.034 | -0.002 | 0.022 | 0.023 | 0.072 | 0.046 | 0.215 | 0.147 | 0.149 |  | 0.200 |
|  |  | **M** | 0.016 | 0.031 | -0.024 | 0.029 | 0.041 | 0.038 | 0.087 | 0.067 | 0.291 | 0.140 | 0.176 | 0.200 |  |
| Life and earth sciences |  |  | **PG** | **NR** | **TI** | **AU** | **IN** | **CU** | **C** | **SC** | **B** | **T** | **F** | **G** | **M** |
|  |  | **PG** |  | **0.613** | 0.117 | 0.035 | 0.113 | 0.117 | 0.176 | 0.161 | -0.030 | -0.014 | -0.011 | -0.023 | -0.050 |
|  |  | **NR** | **0.613** |  | 0.113 | 0.096 | 0.141 | 0.143 | **0.334** | 0.237 | 0.037 | 0.106 | 0.053 | 0.020 | 0.008 |
|  |  | **TI** | 0.117 | 0.113 |  | 0.194 | 0.087 | 0.030 | 0.030 | 0.055 | -0.082 | -0.100 | -0.057 | -0.056 | -0.060 |
|  |  | **AU** | 0.035 | 0.096 | 0.194 |  | **0.519** | 0.280 | 0.198 | 0.187 | 0.027 | 0.087 | 0.055 | 0.016 | 0.032 |
|  |  | **IN** | 0.113 | 0.141 | 0.087 | **0.519** |  | **0.585** | 0.147 | 0.155 | 0.058 | 0.091 | 0.067 | 0.032 | 0.052 |
|  |  | **CU** | 0.117 | 0.143 | 0.030 | 0.280 | **0.585** |  | 0.142 | 0.155 | 0.058 | 0.079 | 0.054 | 0.029 | 0.053 |
|  |  | **C** | 0.176 | **0.334** | 0.030 | 0.198 | 0.147 | 0.142 |  | **0.666** | 0.147 | 0.214 | 0.104 | 0.081 | 0.106 |
|  |  | **SC** | 0.161 | 0.237 | 0.055 | 0.187 | 0.155 | 0.155 | **0.666** |  | 0.093 | 0.106 | 0.055 | 0.047 | 0.072 |
|  |  | **B** | -0.030 | 0.037 | -0.082 | 0.027 | 0.058 | 0.058 | 0.147 | 0.093 |  | 0.281 | 0.262 | 0.263 | **0.365** |
|  |  | **T** | -0.014 | 0.106 | -0.100 | 0.087 | 0.091 | 0.079 | 0.214 | 0.106 | 0.281 |  | **0.350** | 0.189 | 0.203 |
|  |  | **F** | -0.011 | 0.053 | -0.057 | 0.055 | 0.067 | 0.054 | 0.104 | 0.055 | 0.262 | **0.350** |  | 0.190 | 0.226 |
|  |  | **G** | -0.023 | 0.020 | -0.056 | 0.016 | 0.032 | 0.029 | 0.081 | 0.047 | 0.263 | 0.189 | 0.190 |  | 0.262 |
|  |  | **M** | -0.050 | 0.008 | -0.060 | 0.032 | 0.052 | 0.053 | 0.106 | 0.072 | **0.365** | 0.203 | 0.226 | 0.262 |  |
| Mathematics and computer science |  |  | **PG** | **NR** | **TI** | **AU** | **IN** | **CU** | **C** | **SC** | **B** | **T** | **F** | **G** | **M** |
|  |  | **PG** |  | **0.475** | -0.039 | -0.124 | 0.061 | 0.088 | 0.044 | 0.043 | -0.043 | 0.000 | -0.028 | -0.021 | -0.039 |
|  |  | **NR** | **0.475** |  | 0.141 | 0.146 | 0.139 | 0.103 | 0.292 | 0.177 | 0.067 | 0.174 | 0.093 | 0.049 | 0.043 |
|  |  | **TI** | -0.039 | 0.141 |  | 0.194 | 0.045 | -0.026 | 0.110 | 0.077 | -0.016 | 0.025 | 0.020 | -0.010 | -0.009 |
|  |  | **AU** | -0.124 | 0.146 | 0.194 |  | **0.460** | 0.257 | 0.168 | 0.125 | 0.061 | 0.091 | 0.073 | 0.033 | 0.048 |
|  |  | **IN** | 0.061 | 0.139 | 0.045 | **0.460** |  | **0.607** | 0.098 | 0.081 | 0.048 | 0.075 | 0.057 | 0.027 | 0.035 |
|  |  | **CU** | 0.088 | 0.103 | -0.026 | 0.257 | **0.607** |  | 0.081 | 0.082 | 0.035 | 0.045 | 0.036 | 0.019 | 0.027 |
|  |  | **C** | 0.044 | 0.292 | 0.110 | 0.168 | 0.098 | 0.081 |  | **0.665** | 0.098 | 0.126 | 0.079 | 0.062 | 0.070 |
|  |  | **SC** | 0.043 | 0.177 | 0.077 | 0.125 | 0.081 | 0.082 | **0.665** |  | 0.072 | 0.072 | 0.049 | 0.041 | 0.053 |
|  |  | **B** | -0.043 | 0.067 | -0.016 | 0.061 | 0.048 | 0.035 | 0.098 | 0.072 |  | 0.242 | 0.258 | **0.301** | **0.419** |
|  |  | **T** | 0.000 | 0.174 | 0.025 | 0.091 | 0.075 | 0.045 | 0.126 | 0.072 | 0.242 |  | 0.282 | 0.185 | 0.183 |
|  |  | **F** | -0.028 | 0.093 | 0.020 | 0.073 | 0.057 | 0.036 | 0.079 | 0.049 | 0.258 | 0.282 |  | 0.203 | 0.241 |
|  |  | **G** | -0.021 | 0.049 | -0.010 | 0.033 | 0.027 | 0.019 | 0.062 | 0.041 | **0.301** | 0.185 | 0.203 |  | 0.285 |
|  |  | **M** | -0.039 | 0.043 | -0.009 | 0.048 | 0.035 | 0.027 | 0.070 | 0.053 | **0.419** | 0.183 | 0.241 | 0.285 |  |
|  |  |  | **PG** | **NR** | **TI** | **AU** | **IN** | **CU** | **C** | **SC** | **B** | **T** | **F** | **G** | **M** |
| Natural sciences and engineering |  | **PG** |  | **0.457** | 0.032 | -0.092 | 0.064 | 0.089 | 0.089 | 0.076 | -0.046 | 0.039 | 0.014 | 0.004 | -0.034 |
|  |  | **NR** | **0.457** |  | 0.109 | 0.086 | 0.100 | 0.121 | **0.408** | 0.285 | 0.056 | 0.181 | 0.064 | 0.038 | 0.031 |
|  |  | **TI** | 0.032 | 0.109 |  | 0.169 | 0.011 | -0.040 | 0.061 | 0.050 | -0.054 | -0.046 | -0.040 | -0.041 | -0.037 |
|  |  | **AU** | -0.092 | 0.086 | 0.169 |  | **0.437** | 0.248 | 0.172 | 0.163 | 0.043 | 0.048 | 0.023 | 0.011 | 0.042 |
|  |  | **IN** | 0.064 | 0.100 | 0.011 | **0.437** |  | **0.600** | 0.097 | 0.112 | 0.040 | 0.076 | 0.043 | 0.033 | 0.034 |
|  |  | **CU** | 0.089 | 0.121 | -0.040 | 0.248 | **0.600** |  | 0.122 | 0.137 | 0.048 | 0.091 | 0.047 | 0.039 | 0.038 |
|  |  | **C** | 0.089 | **0.408** | 0.061 | 0.172 | 0.097 | 0.122 |  | **0.695** | 0.138 | 0.210 | 0.075 | 0.067 | 0.091 |
|  |  | **SC** | 0.076 | 0.285 | 0.050 | 0.163 | 0.112 | 0.137 | **0.695** |  | 0.090 | 0.141 | 0.050 | 0.046 | 0.064 |
|  |  | **B** | -0.046 | 0.056 | -0.054 | 0.043 | 0.040 | 0.048 | 0.138 | 0.090 |  | 0.194 | 0.158 | 0.194 | 0.289 |
|  |  | **T** | 0.039 | 0.181 | -0.046 | 0.048 | 0.076 | 0.091 | 0.210 | 0.141 | 0.194 |  | 0.242 | 0.143 | 0.166 |
|  |  | **F** | 0.014 | 0.064 | -0.040 | 0.023 | 0.043 | 0.047 | 0.075 | 0.050 | 0.158 | 0.242 |  | 0.131 | 0.176 |
|  |  | **G** | 0.004 | 0.038 | -0.041 | 0.011 | 0.033 | 0.039 | 0.067 | 0.046 | 0.194 | 0.143 | 0.131 |  | 0.172 |
|  |  | **M** | -0.034 | 0.031 | -0.037 | 0.042 | 0.034 | 0.038 | 0.091 | 0.064 | 0.289 | 0.166 | 0.176 | 0.172 |  |
| Social sciences and humanities |  |  | **PG** | **NR** | **TI** | **AU** | **IN** | **CU** | **C** | **SC** | **B** | **T** | **F** | **G** | **M** |
|  |  | **PG** |  | **0.671** | 0.174 | 0.103 | 0.127 | 0.106 | 0.173 | 0.100 | -0.016 | 0.051 | -0.007 | -0.015 | -0.021 |
|  |  | **NR** | **0.671** |  | **0.312** | **0.327** | 0.264 | 0.173 | **0.397** | 0.260 | 0.059 | 0.201 | 0.073 | 0.045 | 0.033 |
|  |  | **TI** | 0.174 | **0.312** |  | **0.343** | 0.216 | 0.092 | 0.209 | 0.156 | 0.008 | 0.107 | 0.033 | 0.003 | 0.009 |
|  |  | **AU** | 0.103 | **0.327** | **0.343** |  | **0.670** | **0.368** | **0.419** | **0.322** | 0.100 | 0.247 | 0.111 | 0.075 | 0.077 |
|  |  | **IN** | 0.127 | 0.264 | 0.216 | **0.670** |  | **0.590** | **0.319** | 0.244 | 0.078 | 0.177 | 0.080 | 0.053 | 0.058 |
|  |  | **CU** | 0.106 | 0.173 | 0.092 | **0.368** | **0.590** |  | 0.200 | 0.170 | 0.052 | 0.096 | 0.045 | 0.038 | 0.043 |
|  |  | **C** | 0.173 | **0.397** | 0.209 | **0.419** | **0.319** | 0.200 |  | **0.623** | 0.160 | 0.278 | 0.125 | 0.100 | 0.109 |
|  |  | **SC** | 0.100 | 0.260 | 0.156 | **0.322** | 0.244 | 0.170 | **0.623** |  | 0.113 | 0.173 | 0.078 | 0.072 | 0.085 |
|  |  | **B** | -0.016 | 0.059 | 0.008 | 0.100 | 0.078 | 0.052 | 0.160 | 0.113 |  | 0.239 | 0.222 | 0.242 | **0.338** |
|  |  | **T** | 0.051 | 0.201 | 0.107 | 0.247 | 0.177 | 0.096 | 0.278 | 0.173 | 0.239 |  | **0.302** | 0.177 | 0.166 |
|  |  | **F** | -0.007 | 0.073 | 0.033 | 0.111 | 0.080 | 0.045 | 0.125 | 0.078 | 0.222 | **0.302** |  | 0.222 | 0.215 |
|  |  | **G** | -0.015 | 0.045 | 0.003 | 0.075 | 0.053 | 0.038 | 0.100 | 0.072 | 0.242 | 0.177 | 0.222 |  | 0.253 |
|  |  | **M** | -0.021 | 0.033 | 0.009 | 0.077 | 0.058 | 0.043 | 0.109 | 0.085 | **0.338** | 0.166 | 0.215 | 0.253 |  |
