# Supplementary material for: Characterizing Social Media Metrics of Scholarly Papers: The Effect of Document Properties and Collaboration Patterns
Source: PLoS One. 2015 Mar 17;10(3):e0120495. doi: 10.1371/journal.pone.0120495 (PMC4363625; doi:10.1371/journal.pone.0120495)
Supplement: S2 Table — Equations for linear regressions based on citation (C) and social media density (B, T, F, G, M) shown in Figs. 2–5 and S2 and S3 Figs. Positive and negative slopes with β≥±0.001as well as coefficients of determination R2≥0.300 are highlighted in bold. (DOCX) [file pone.0120495.s005.docx]

**S2 Table. Linear regression equations per variables and LR fields.** Equations for linear regressions based on citation (C) and social media density (B, T, F, G, M) shown in Figs. 2-5 and S2 and S3 Figs. Positive and negative slopes with β≥±0.001as well as coefficients of determination R^2^≥0.300 are highlighted in bold.

| **LR field** |  | |  | **C** | **B** | **T** | **F** | **G** | **M** |
| --- | --- | --- | --- | --- | --- | --- | --- | --- | --- |
| All fields | | **PG** | | y = **0.0433**x + 2.6242  **R² = 0.361** | y = -0.0003x + 0.0323  R² = 0.218 | y = **-0.0056**x + 0.7822  R² = 0.250 | y = -0.0003x + 0.0767  R² = 0.025 | y = -4E-05x + 0.0122  R² = 0.023 | y = -0.0003x + 0.0156  R² = 0.267 |
|  |  | **NR** | | y = **0.0622**x + 0.8474  **R² = 0.934** | y = 0.0005x + 0.0094  **R² = 0.786** | y = **0.0111**x + 0.3275  **R² = 0.803** | y = **0.0015**x + 0.0269  **R² = 0.661** | y = 0.0002x + 0.0046  **R² = 0.595** | y = 0.0002x + 0.0045  **R² = 0.458** |
|  |  | **TI** | | y = **0.0046**x + 3.2079  R² = 0.234 | y = -0.0002x + 0.0485  **R² = 0.618** | y = **-0.0021**x + 1.0118  **R² = 0.365** | y = -6E-05x + 0.0951  R² = 0.008 | y = -0.0001x + 0.0253  **R² = 0.380** | y = -6E-05x + 0.0169  **R² = 0.367** |
|  |  | **AU** | | y = **0.4876**x + 0.8889  **R² = 0.964** | y = **0.0062**x - 0.0055  **R² = 0.895** | y = **0.1413**x + 0.0089  **R² = 0.918** | y = **0.0153**x + 0.0062 **R² = 0.934** | y = **0.0021**x + 0.0001  **R² = 0.721** | y = **0.0038**x - 0.0105  **R² = 0.822** |
|  |  | **IN** | | y = **0.7978**x + 1.4793  **R² = 0.965** | y = **0.0143**x - 0.0095  **R² = 0.819** | y = **0.282**x + 0.1273  **R² = 0.904** | y = **0.0305**x + 0.016  **R² = 0.936** | y = **0.0042**x + 0.0036  **R² = 0.720** | y = **0.007**x - 0.0056  **R² = 0.717** |
|  |  | **CU** | | y = **1.5554**x + 1.3358  **R² = 0.974** | y = **0.0231**x + 0.0062  **R² = 0.900** | y = **0.5051**x + 0.0014  **R² = 0.953** | y = 0.0466x + 0.0234  **R² = 0.871** | y = **0.0078**x + 0.0062  **R² = 0.876** | y = **0.0137**x - 0.0056  **R² = 0.895** |
| Biomedical and health sciences |  | | **PG** | y = **0.2089**x + 2.1745  **R² = 0.826** | y = 0.0007x + 0.0326  R² = 0.154 | y = **0.0287**x + 0.9937  **R² = 0.324** | y = **0.0075**x + 0.0659  R² = 0.214 | y = 0.0001x + 0.0166  R² = 0.015 | y = -6E-05x + 0.0156  R² = 0.005 |
|  |  | | **NR** | y = **0.0674**x + 1.1446  **R² = 0.927** | y = 0.0006x + 0.011  **R² = 0.808** | y = **0.0159**x + 0.6461  **R² = 0.776** | y = **0.0026**x + 0.0523  **R² = 0.555** | y = 0.0003x + 0.0043  **R² = 0.617** | y = 0.0002x + 0.0052  **R² = 0.511** |
|  |  | | **TI** | y = **-0.001**x + 4.5345  R² = 0.003 | y = -0.0002x + 0.059  **R² = 0.601** | y = **-0.0048**x + 1.7789  **R² = 0.465** | y = -0.0004x + 0.1996  R² = 0.095 | y = -0.0001x + 0.0305  R² = 0.281 | y = -0.0001x + 0.0305  R² = 0.281 |
|  |  | | **AU** | y = **0.472**x + 1.2663 **R² = 0.901** | y = **0.0054**x + 0.0007  **R² = 0.805** | y = **0.125**x + 0.5209 **R² = 0.805** | y = **0.0131**x + 0.0714  **R² = 0.847** | y = **0.0015**x + 0.0068  **R² = 0.591** | y = **0.0032**x - 0.0069  **R² = 0.792** |
|  |  | | **IN** | y = **0.7882**x + 1.8309  **R² = 0.947** | y = **0.0111**x + 0.0039  **R² = 0.924** | y = **0.2629**x + 0.6728  **R² = 0.921** | y = **0.0303**x + 0.0731  **R² = 0.915** | y = **0.0029**x + 0.0103  **R² = 0.674** | y = **0.0048**x + 0.0029  **R² = 0.617** |
|  |  | | **CU** | y = **1.6026**x + 2.2914  **R² = 0.951** | y = **0.0204**x + 0.0235  **R² = 0.689** | y = **0.5867**x + 0.6235  **R² = 0.875** | y = **0.0553**x + 0.1001  **R² = 0.781** | y = **0.0062**x + 0.0149  **R² = 0.634** | y = **0.0115**x + 0.006  **R² = 0.778** |
| Life and earth sciences |  | | **PG** | y = **0.0888**x + 2.5587  **R² = 0.836** | y = **-0.0013**x + 0.0796  R² = 0.170 | y = **-0.0338**x + 1.5161  **R² = 0.430** | y = **-0.0014**x + 0.122  R² = 0.175 | y = -0.0007x + 0.0291  **R² = 0.390** | y = **-0.0013**x + 0.0489  R² = 0.221 |
|  |  | | **NR** | y = **0.0508**x + 1.1195  **R² = 0.927** | y = 0.0005x + 0.0308  **R² = 0.350** | y = **0.0061**x + 0.7219  R² = 0.244 | y = **0.0012**x + 0.0539  **R² = 0.419** | y = 0.0002x + 0.0101  R² = 0.254 | y = 0.0001x + 0.0165  R² = 0.071 |
|  |  | | **TI** | y = **-0.0112**x + 5.0146  **R² = 0.371** | y = -0.0008x + 0.1449  **R² = 0.457** | y = **-0.0124**x + 2.4741  **R² = 0.552** | y = -0.0008x + 0.2012  **R² = 0.374** | y = -0.0004x + 0.0667  **R² = 0.310** | y = -0.0003x + 0.0605  **R² = 0.324** |
|  |  | | **AU** | y = **0.5291**x + 0.7587  **R² = 0.944** | y = **0.0135**x - 0.0201  **R² = 0.846** | y = **0.2176**x - 0.1255  **R² = 0.872** | y = **0.0218**x - 0.0018  **R² = 0.832** | y = **0.0044**x - 0.0049  **R² = 0.727** | y = **0.0115**x - 0.0452 **R² = 0.659** |
|  |  | | **IN** | y = **0.9835**x + 0.8098  **R² = 0.906** | y = **0.0335**x - 0.045  **R² = 0.826** | y = **0.4431**x - 0.1448  **R² = 0.939** | y = **0.0393**x + 0.0268  **R² = 0.825** | y = **0.0088**x - 0.0038  **R² = 0.695** | y = **0.027**x - 0.063  **R² = 0.708** |
|  |  | | **CU** | y = **1.9333**x + 0.0285 **R² = 0.866** | y = **0.0493**x - 0.0034  **R² = 0.666** | y = **0.9986**x - 1.0608  **R² = 0.924** | y = **0.057**x + 0.0603  **R² = 0.613** | y = **0.0133**x + 0.0071  **R² = 0.560** | y = **0.0344**x - 0.022  **R² = 0.730** |
| Mathematics and computer science |  | | **PG** | y = 0.0006x + 1.5669  R² = 0.000 | y = -0.0006x + 0.0209  R² = 0.237 | y = **-0.0122**x + 0.4722  R² = 0.292 | y = -0.0009x + 0.0359  R² = 0.275 | y = -0.0003x + 0.0122  R² = 0.094 | y = -0.0003x + 0.0102  R² = 0.238 |
|  |  | | **NR** | y = **0.0426**x + 0.4906  **R² = 0.941** | y = 0.0007x - 0.004  **R² = 0.618** | y = **0.0179**x - 0.1242  **R² = 0.733** | y = **0.0015**x - 0.0114  **R² = 0.711** | y = 0.0003x + 4E-05  R² = 0.240 | y = 0.0003x - 0.0008  **R² = 0.350** |
|  |  | | **TI** | y = **0.0022**x + 1.5783  R² = 0.032 | y = -0.0001x + 0.0249  **R² = 0.383** | y = 0.0006x + 0.352  R² = 0.001 | y = -6E-05x + 0.047  R² = 0.001 | y = -9E-05x + 0.0184  R² = 0.077 | y = -5E-05x + 0.0098  R² = 0.255 |
|  |  | | **AU** | y = **0.4328**x + 0.1742  **R² = 0.753** | y = **0.0178**x - 0.065  **R² = 0.761** | y = **0.2373**x - 0.5255  **R² = 0.873** | y = **0.0213**x - 0.0509  **R² = 0.553** | y = **0.0068**x - 0.0226  **R² = 0.334** | y = **0.0089**x - 0.0323 **R² = 0.786** |
|  |  | | **IN** | y = **1.575**x - 3.4156 **R² = 0.378** | y = **0.0601**x - 0.1707 R² = 0.252 | y = **1.0128**x - 2.8079  **R² = 0.404** | y = **0.067**x - 0.1055  **R² = 0.435** | y = **0.0333**x - 0.111 R² = 0.267 | y = **0.0328**x - 0.0929 R² = 0.186 |
|  |  | | **CU** | y = **5.8188**x - 12.236  **R² = 0.408** | y = **0.3208**x - 0.869  **R² = 0.445** | y = **4.7887**x - 12.978  **R² = 0.498** | y = **0.1923**x - 0.2509  **R² = 0.151** | y = **0.1447**x - 0.4235  **R² = 0.544** | y = **0.1524**x - 0.3795  **R² = 0.480** |
| Natural sciences and engineering |  | | **PG** | y = **0.2252**x + 1.4392  **R² = 0.837** | y = -0.0006x + 0.0398  R² = 0.038 | y = **-0.0032**x + 0.5345  R² = 0.005 | y = 0.0004x + 0.0398  R² = 0.015 | y = 0.0004x + 0.0115  R² = 0.067 | y = -0.0007x + 0.0245  R² = 0.145 |
|  |  | | **NR** | y = **0.0894**x + 0.5065  **R² = 0.968** | y = 0.0005x + 0.0066  **R² = 0.670** | y = **0.0073**x + 0.0673  **R² = 0.750** | y = 0.0006x + 0.0106  **R² = 0.691** | y = 0.0002x + 0.0036  **R² = 0.306** | y = 0.0002x + 0.005  **R² = 0.301** |
|  |  | | **TI** | y = **-0.0054**x + 4.8808  R² = 0.078 | y = -0.0004x + 0.0683  **R² = 0.512** | y = **-0.0038**x + 0.8586  R² = 0.190 | y = -0.0007x + 0.1227  R² = 0.051 | y = -0.0003x + 0.0458  R² = 0.194 | y = -0.0002x + 0.031  **R² = 0.511** |
|  |  | | **AU** | y = **0.3708**x + 2.2106  **R² = 0.956** | y = **0.0105**x - 0.0272  **R² = 0.907** | y = **0.1027**x - 0.1748  **R² = 0.821** | y = **0.0101**x - 0.0167  **R² = 0.795** | y = **0.0043**x - 0.0135  **R² = 0.442** | y = **0.0096**x - 0.0423  **R² = 0.628** |
|  |  | | **IN** | y = **0.7548**x + 2.3385  **R² = 0.907** | y = **0.0311**x - 0.0655  **R² = 0.515** | y = **0.2977**x - 0.5774 **R² = 0.461** | y = **0.0194**x - 0.0023  **R²** **= 0.607** | y = **0.012**x - 0.02  **R² = 0.517** | y = **0.0201**x - 0.0452  **R² = 0.557** |
|  |  | | **CU** | y = **1.4**x + 1.8097  **R² = 0.920** | y = **0.0342**x - 0.0282  **R² = 0.850** | y = **0.355**x - 0.3194  **R² = 0.760** | y = **0.0206**x + 0.0126  **R² = 0.741** | y = **0.0136**x - 0.0066  **R² = 0.783** | y = **0.0265**x - 0.0431  **R² = 0.739** |
| Social sciences and humanities |  | | **PG** | y = **-0.0071**x + 1.6998  R² = 0.024 | y = **-0.0041**x + 0.1332  **R² = 0.356** | y = **-0.0743**x + 2.8014  **R² = 0.474** | y = **-0.0055**x + 0.2021  **R² = 0.527** | y = **-0.002**x + 0.0603  **R² = 0.467** | y = **-0.0019**x + 0.0562  **R² = 0.304** |
|  |  | | **NR** | y = **0.025**x + 0.5581  **R² = 0.865** | y = 0.0003x + 0.0381  R² = 0.097 | y = **0.0104**x + 1.0604  R² = 0.170 | y = **0.0012**x + 0.0589  R² = 0.219 | y = 7E-05x + 0.0207  R² = 0.014 | y = 0.0001x + 0.0109  R² = 0.091 |
|  |  | | **TI** | y = **0.005**x + 1.3039  R² = 0.199 | y = -0.0002x + 0.0688  R² = 0.049 | y = **-0.0036**x + 1.7381  R² = 0.106 | y = -0.0003x + 0.1187  R² = 0.038 | y = -0.0002x + 0.037  **R² = 0.309** | y = -8E-05x + 0.0214  R² = 0.083 |
|  |  | | **AU** | y = **0.4937**x - 0.0477  **R² = 0.767** | y = **0.0297**x - 0.069  **R² = 0.635** | y = **0.3811**x + 0.6502  **R² = 0.731** | y = **0.0417**x + 0.0053  **R² = 0.733** | y = **0.0128**x - 0.0322  **R² = 0.425** | y = **0.028**x - 0.127  **R² = 0.673** |
|  |  | | **IN** | y = **0.6166**x + 0.4704  **R² = 0.511** | y = **0.0308**x + 0.0307 **R² = 0.434** | y = **0.452**x + 1.3586 **R² = 0.524** | y = **0.0593**x + 0.0392  **R² = 0.526** | y = **0.0071**x + 0.0333  R² = 0.184 | y = **0.027**x - 0.0246  R² = 0.268 |
|  |  | | **CU** | y = **0.7685**x + 1.6754  **R² = 0.558** | y = **0.0289**x + 0.1421  R² = 0.124 | y = **0.3714**x + 3.1307  R² = 0.109 | y = **0.0335**x + 0.2933  R² = 0.068 | y = **0.0029**x + 0.0841  R² = 0.008 | y = **0.0232**x + 0.0856  R² = 0.127 |
